# Evolutionary History of Tissue Kallikreins

**DOI:** 10.1371/journal.pone.0013781

**Published:** 2010-11-01

**Authors:** Athanasia Pavlopoulou, Georgios Pampalakis, Ioannis Michalopoulos, Georgia Sotiropoulou

**Affiliations:** 1 Department of Pharmacy, School of Health Sciences, University of Patras, Rion-Patras, Greece; 2 Biomedical Research Foundation of the Academy of Athens, Athens, Greece; American Museum of Natural History, United States of America

## Abstract

The gene family of human kallikrein-related peptidases (*KLKs*) encodes proteins with diverse and pleiotropic functions in normal physiology as well as in disease states. Currently, the most widely known KLK is KLK3 or prostate-specific antigen (PSA) that has applications in clinical diagnosis and monitoring of prostate cancer. The *KLK* gene family encompasses the largest contiguous cluster of serine proteases in humans which is not interrupted by non-*KLK* genes. This exceptional and unique characteristic of KLKs makes them ideal for evolutionary studies aiming to infer the direction and timing of gene duplication events. Previous studies on the evolution of KLKs were restricted to mammals and the emergence of *KLKs* was suggested about 150 million years ago (mya). In order to elucidate the evolutionary history of KLKs, we performed comprehensive phylogenetic analyses of KLK homologous proteins in multiple genomes including those that have been completed recently. Interestingly, we were able to identify novel reptilian, avian and amphibian KLK members which allowed us to trace the emergence of KLKs 330 mya. We suggest that a series of duplication and mutation events gave rise to the *KLK* gene family. The prominent feature of the KLK family is that it consists of tandemly and uninterruptedly arrayed genes in all species under investigation. The chromosomal co-localization in a single cluster distinguishes KLKs from trypsin and other trypsin-like proteases which are spread in different genetic loci. All the defining features of the KLKs were further found to be conserved in the novel KLK protein sequences. The study of this unique family will further assist in selecting new model organisms for functional studies of proteolytic pathways involving KLKs.

## Introduction

Human tissue kallikrein-related serine peptidases (KLKs) constitute a single family of 15 highly conserved trypsin- or chymotrypsin-like serine proteases encoded by the largest contiguous cluster of protease-encoding genes (*KLK1-15*) in the human genome mapped to chromosomal locus 19q13.4 [1]. The most widely known member of the KLK family is KLK3 or PSA (prostate-specific antigen) that has applications in the diagnosis and monitoring of prostate cancer [2]. The *KLK* contiguous cluster is not interrupted by other non-*KLK* genes, an additional feature that makes this family unique. Collectively, all the above characteristics establish the KLK family as a family of great importance for evolutionary studies. Tissue KLKs are usually divided into two groups the “classical” and the “non-classical” KLKs. The term “classical” KLKs is referred to the first members of the human KLK family that were identified, namely KLK1, KLK2, and KLK3 (PSA), whereas the rest are often referred to as “non-classical” [1], [3].

All currently reported *KLK* genes encode for single-chain prepro-enzymes with lengths varying between 244 and 293 amino acid residues and approximately share 40% protein identity. The preproKLKs are proteolytically processed to enzymatically inactive proKLKs that are secreted *via* the removal of an amino-terminal signal peptide. Subsequently, proKLKs are activated to mature peptidases extracellularly by specific proteolytic cleavage of their amino-terminal propeptide, a key step in the regulation of KLK function [1], [4]–[5]. Characteristic features of KLKs are the invariant residues of the active-site catalytic triad His^57^, Asp^102^ and Ser^195^, as well as a conserved Gly^193^ (human chymotrypsin numbering system) which is implicated in stabilizing the oxyanion intermediate of the internal peptide bond during hydrolysis [6].

KLKs are expressed in a wide variety of tissues including the pancreas, heart, lung, central nervous system, salivary glands and endocrine-regulated tissues such as thyroid, breast, testis, ovary, prostate, indicating that they participate in important biological processes [7], [8]. Indeed, several lines of evidence support that KLKs cooperate in complex proteolytic cascade pathways to regulate physiological and pathological processes [5], [9]. For instance, KLK5, KLK7 and KLK14 are involved in skin desquamation and other skin diseases [10]–[13] while KLK2, KLK3 and KLK5 have been involved in seminal plasma liquefaction [5], [9]. Of particular note, KLKs are implicated in different stages of cancer development and progression and have emerged as powerful tumor markers as demonstrated by the PSA testing [1].

Previous efforts focused on the evolutionary history of KLKs focused in the characterization of the mouse [14], [15], rat [16], [17] and pig genes [18], as well as of individual members in mastomys [19], cynomolgus monkey [20], rhesus monkey [21], [22], dog [23], guinea pig [24], macaque orangutan, chimpanzee, gorilla [25], cat [26], horse and cow [17], [22] and cotton-top tamarin [27]. Elliot et al. [28] had performed the first Bayesian phylogenetic analysis and suggested the origin of the KLK family before the marsupial-placental split (approximately 125–175 million years ago, mya). An additional advantage in evolution studies has emerged based on the huge number of sequences deposited in the public databases and the availability of an increasing number of sequenced genomes, as well as the availability of computational tools for crossgenome analyses.

In the present study, a comprehensive phylogenetic analysis of the KLK proteins was performed employing a maximum likelihood-based method in order to unravel the evolutionary history of the KLK family. Interestingly, three reptilian, two avian and one amphibian KLK homologues were detected which allowed us to trace the evolutionary origin of KLKs earlier than it was previously thought, approximately 330 mya. Primary sequences, as well as the predicted secondary and tertiary structures of putative KLK peptide sequences were analyzed. The genomic organization of the KLK genes was further examined and it was shown that in different species these genes cluster together at syntenic loci. Collectively, we suggest that KLKs are also present in non-therian species covering an evolutionary distance from amphibia to eutheria and they cluster at a single locus.

## Results

### Identification of KLK homologous proteins

The complete or almost complete genomes of species representing major taxonomic divisions (according to the NCBI taxonomy database) [29] were searched for putative KLK protein sequences. Collectively, 260 KLK homologous protein sequences were identified in 26 species, as follows: primates (78), rodentia (57), carnivore (24), insectivore (9), perissodactyla (12), cetartiodactyla (23), chiroptera (12), afrotheria (15), xenarthra (5), metatheria (14), prototheria (5), sauria (3), aves (2), amphibia (1), pisces (0), ascidia (0) and insect (0).

**Figure 1:**
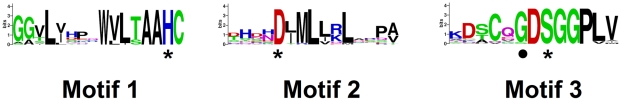
Sequence logo of the three catalytic motifs present in KLKs. The number of each of the motifs is indicated below. The height of each letter is proportional to the frequency of the corresponding residue at that position, and the letters are ordered so the most frequent is on top. The conserved catalytic triad residues are indicated with asterisks. The dot indicates the conserved glycine residue present in the oxyanion hole.

**Figure 2:**
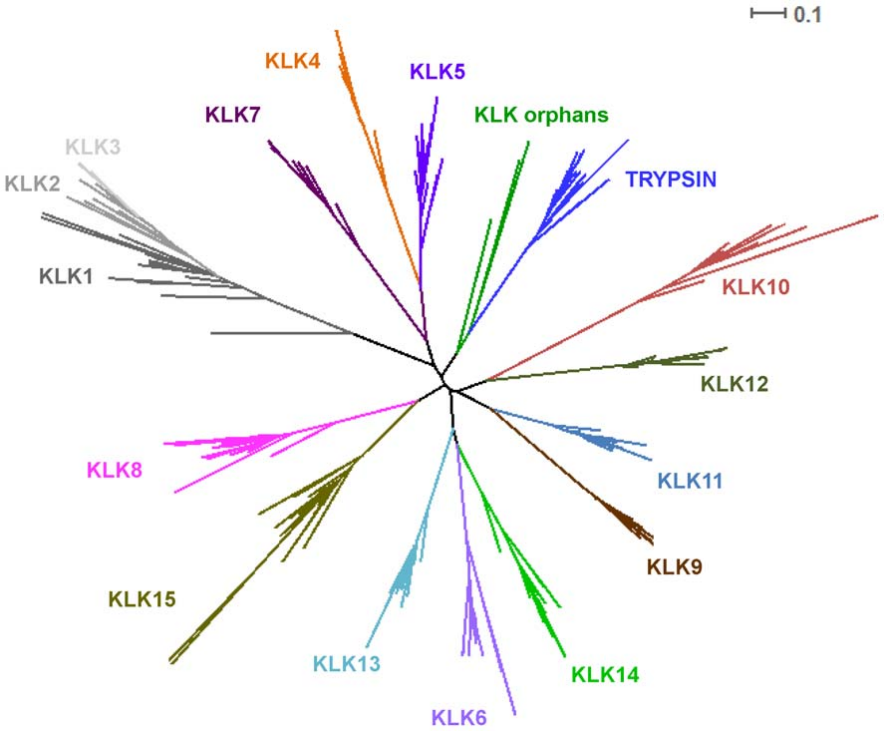
Maximum likelihood tree based on the core domain of the KLK homologues. The support values (>50%) are indicated at the nodes. The branch lengths depict evolutionary distance. The trypsin proteins are used as outgroup. The scale bar at the upper right denotes evolutionary distance of 0.1 amino acids per position.

**Figure 3:**
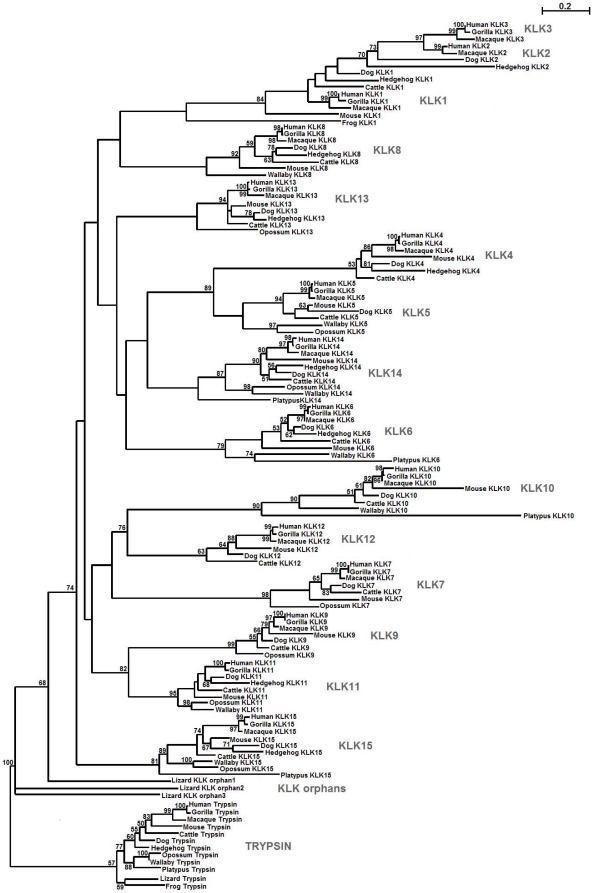
Representative maximum likelihood phylogenetic tree of KLK homologues. Bootstrap values (>50%) are indicated at the nodes. The branch lengths depict evolutionary distance. The trypsin proteins are used as outgroup. The scale bar at the upper right denotes evolutionary distance of 0.2 amino acids per position.

**Figure 4:**
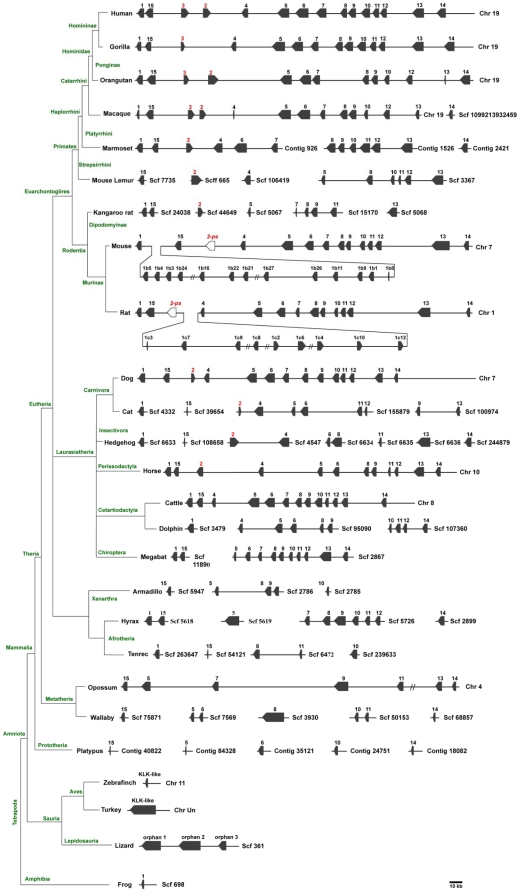
Schematic representation of the chromosomal arrangement of *KLKs*. The orientation and approximate position and size of the kallikrein genes. The *KLK* encoding genes are shown as filled arrowheads, whereas the *KLK* pseudogenes are represented by open arrowheads. The *KLK2, KLK2-ps* and *KLK3* genes are indicated by red. Loci are drawn in approximate scale. On the left, a NCBI taxonomy-based cladogram shows the evolutionary relationships of taxa and the taxonomic classes.

All identified sequences are depicted in Table S1. As shown in Figure 1, the amino acid residues corresponding to the active site residues (chymotrypsin numbering: His^57^, Asp^102^ and Ser^195^) are conserved among species. The core trypsin domain of the 260 retrieved sequences was used to reconstruct a maximum likelihood-based phylogenetic tree (Figure 2). The recently released zebrafinch and platypus genomes [30], [31] allowed us also to identify novel partial KLK-like sequences (zebra finch) (Table S1) which were not included in the phylogenetic analysis because they would decrease the resolution of the phylogenetic tree. In this tree, 13 coherent monophyletic branches were identified which permitted the preliminary classification of the candidate KLK protein sequences into 13 groups (Figure 2). Subsequently, a sample of 87 KLK protein sequences was chosen for more accurate phylogenetic analysis using the maximum likelihood method (Figure 3). This selection was done based on representative taxa (from the main taxonomic divisions). The generated tree is overall well-supported (Figure 3). The low bootstrap values in some deep-branching nodes suggest alternative branching. In the inferred phylogenetic tree, 13 highly resolved clades are distinguished which correspond to the classical KLKs (KLK1 to KLK3) and the other 12 KLK members (KLK4 to KLK15) (Figure 3). Interestingly, three reptilian KLK-related sequences appear to form their own separate clades, with relatively high support values, and they were arbitrarily referred to as “orphan KLKs” (Figures 2 and 3). Importantly, examination of the chromosomal localization of the *KLK* genes in different species reveals that the position and orientation of these genes are highly preserved (Figure 4). In addition, as is demonstrated in Figure S1 the splicing patterns are consistent between all KLK sequences, and the amino acid residues encompassing the active site are located in different exons as in human *KLK* genes.

### Conservation of KLK defining structural features in KLK homologous proteins

As mentioned previously, the identified KLK homologues contain the invariant residues of the active-site catalytic triad (Figure 1). However, the conserved glycine residue of motif 3 which is highly conserved in serine proteases is not conserved in KLK10 orthologues. This discrepancy is due to the fact that the KLK10 homologues contain a Gly193Ser substitution [6]. Platypus is an exception however, being the only animal found to have conserved Gly193 in KLK10, probably indicating that this mutation was later introduced in order for the protein to acquire a very strict specificity and likely a highly specific biological function. Interestingly, a recent study failed to demonstrate enzymatic activity for KLK10 against synthetic substrates, suggesting that either KLK10 is inactive or it is highly specific for a single substrate, yet to be identified [32]. Mouse KLK13 points to the later direction since, although it has the Gly193Asp mutation, it possesses enzymatic activity [33]. Another exception is the presence of mutation at Asp102Ala (chymotrypsin numbering) in KLK2 of Rhesus monkey (*Macaca mulata*). This mutation renders the enzyme inactive, since Asp102 is a catalytic residue.

**Figure 5:**
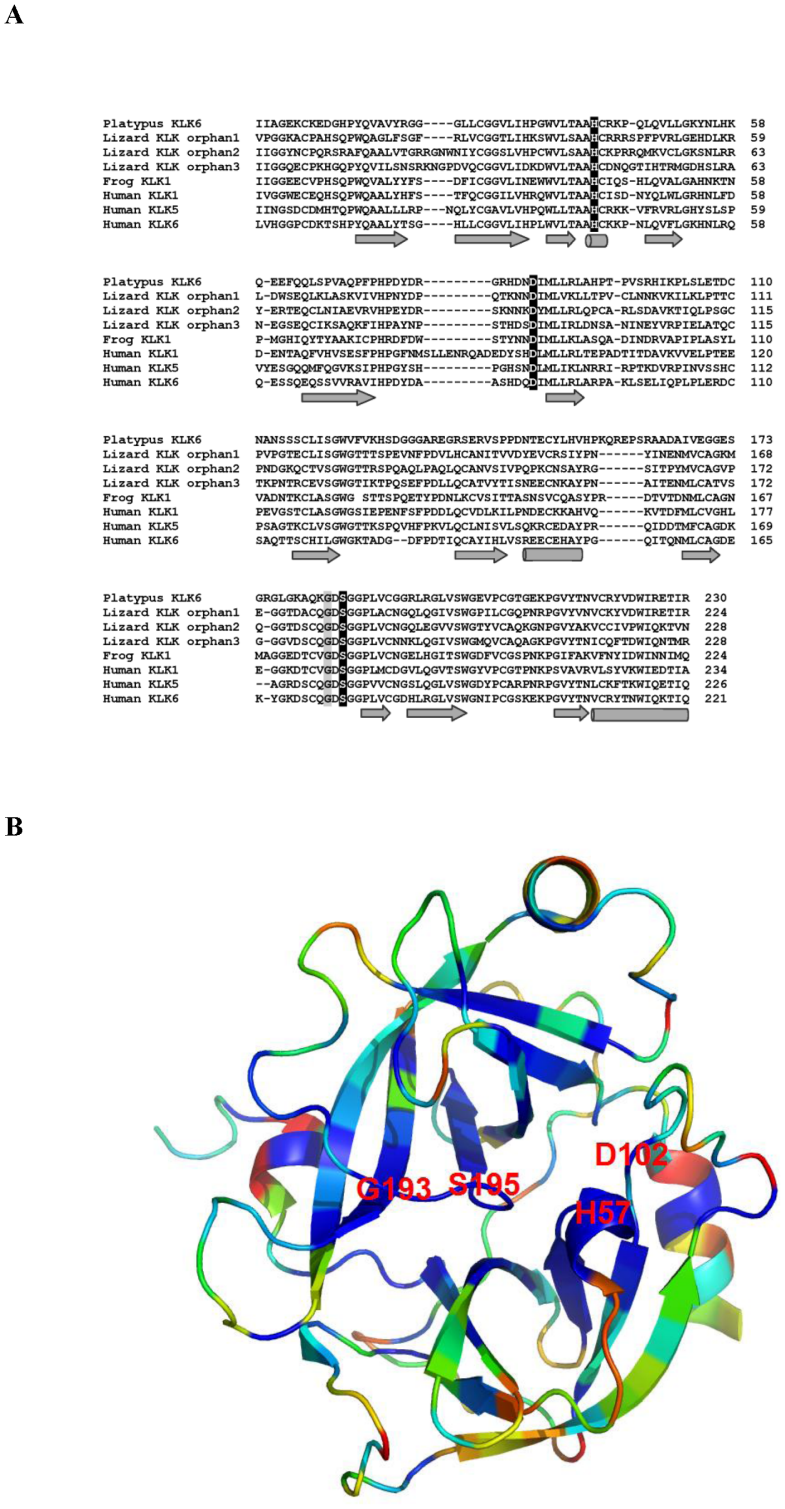
A, Secondary structure prediction of putative KLKs. Sequences corresponding to the conserved trypsin domain were aligned using PROMALS3D. The residues of the catalytic triad are shown on the black background; the glycine residue is shown on the gray background. The consensus secondary structure is shown at the bottom, where the *α*-helices are represented by cylinders and the *β*-sheets by arrows. KLKs with known crystal structure were used as reference; their PDB accession codes are: KLK1(1spj), KLK5(2psy), KLK6 (1gvl). **B, Tertiary structure of proKLK6.** Graphic visualization of the human proKLK6 protein color coded by conservation score, where the region in blue corresponds to the most highly conserved region. KLK6 is represented as a cartoon. The four conserved amino acid residues (His ^57^, Asp^102^, Ser^195^ and Gly^193^) are indicated.

Furthermore, it is demonstrated that the novel KLK amino acid sequences possess the secondary structure of known KLKs [34]–[36] (Figure 5a). The degree of conservation of the four catalytically important amino acids is shown in the known three dimensional structure of human proKLK6 [34] (Figure 5b) where they appear to be located in the most conserved region which is the cleft between the two trypsin-like serine protease domains (thrombin, subunit H beta-barrels; CATH Code: 2.40.10.10).

### Organization of the KLK cluster

The exponential accumulation of genomic sequences allowed us to study the evolution of the *KLK* gene family in different species. As shown in Figure 4 and mentioned previously, the putative KLK proteases are encoded by uninterrupted, contiguous clusters of genes-at least in the most complete genomes - suggesting a preserved standard sequential order. This co-clustering of KLKs at a single locus is opposed to the other multigene peptide families where the paralogous genes are scattered on a one or multiple chromosomes [37] . However, due to incomplete genomic studies, *KLK* genes of several species [31] are ‘dispersed’ in different genomic scaffolds (Figure 4). For this reason, a KLK member is considered to be absent only if the gene is not detected and at the same time the *KLK* genes that flank it in the prototypical sequential order (1) are detected in the same chromosome/scaffold/contig. The two *KLK2* pseudogenes (*KLK2-ps*) found in murinae [17] were included in the analysis in order to enhance our understanding regarding the evolution of the *KLK2* gene.

### Phylogenetic analyses of the KLK family

The two phylogenetic trees (Figures 2 and 3), reconstructed using the maximum likelihood method, are congruent with similar topologies. These phylogenies suggest that, apart from the fifteen “conventional” KLK family members, three ‘orphan’ KLKs are present in anole lizard. The lizard KLK orphans appear to arise from the basal node (Figure 4) leading to the suggestion that they are the members of the KLK family that diverged earliest (“proto-KLKs”). There are three lines of evidence which suggest that these are true KLKs: (a) true KLK hits were yielded in a reciprocal BLAST, (b) a lizard trypsin exists which clusters with the fellow trypsins (Figure 3), (c) the lizard *KLK* genes are arranged in tandem repeats in a single genomic scaffold (Figure 4). KLK6, KLK14 and KLK15 were detected for first time in Prototheria (Figure 4). Besides, KLK15 is present in all organisms from Prototheria up to humans (Figure 4). KLK7, KLK8 and KLK13 apparently arrived later in the KLK family since they were both detected first in metatheria (Figure 4). Regarding KLK1, it was first detected in amphibia as a *bona fide* KLK and then appeared again in afrotheria whereas KLK2 was detected in laurasiatheria for the first time (Figure 4). We propose that the *KLK2* gene is the result of the duplication/inversion of the *KLK1* gene in an early laurasiatherian mammal. The findings above are in agreement with a previously proposed hypothesis [38], [39] for eutherian evolution. According to this hypothesis xenarthra and afrotheria are sister groups -with xenarthra being the more ancient- placed at a basal position relative to the laurasiatheria and euarchontoglires. The presence of KLK2 in carnivora, insectivora and perissodactyla and its absence in cetartiodactyla and chiroptera (Figure 4) triggers that speculation that a *KLK2* gene may have existed in these species which was either deleted or arose later in laurasiatherian evolution. In such an event the *KLK2* gene was inactivated later in the course of evolution in the murine lineage resulting to a *KLK2* pseudogene (Figure 4). Also, in gorilla, the *KLK2* gene must have been deleted in the course of evolution, since it has been reported to have only exons I and V and we were also unable to identify the gene [40]. Instead, several duplications of the *KLK1* gene occurred later in the evolution yielding 13 *KLK1* homologues in the mouse genome and 9 *KLK1* homologues in the rat genome (Figure 4). Both the inactivation of *KLK2* and the series of *KLK1* duplication events apparently occurred after the divergence of the murine from other rodents such as the kangaroo rat since a functional KLK2 exists and no duplication of *KLK1* was observed in its genome.

The *KLK2* gene maintains the same orientation in all genomes except in perissodactyla suggesting that the direction of *KLK2* transcription differs from species to species. Although, the equine KLK2 has a predicted chymotrypsin-like specificity similar to that of KLK3 [41], it shares the highest degree of sequence identity with KLK2 (data not shown), thus the symbol KLK2 was assigned to this protein. The canine KLK2 enzyme, though, was found to display proteolytic specificity similar to that of KLK2 but not KLK3 [17]. We propose a duplication event of *KLK2* which produced a *KLK3* in catarrhini. Both KLK2 and KLK3 enzymes are secreted by the prostate gland where the zymogen KLK3 was shown to be activated by KLK2 [42]. The presence of these two enzymes in humans, apes and Old World monkeys (Figure 4) leads to the suggestion that these enzymes are involved in physiological processes that are specific to catarrhini primates as outlined later. The classical KLKs form their own separate clade that is highly supported (Figure 2 and 3). The monophyletic groups KLK9 and KLK11 appear to have strong homology as confirmed by relatively high bootstrap values (Figures 2 and 3), triggering the speculation that tandem duplication events, apparently before the marsupial-placental divergence, may have copied *KLK9* and *KLK11*. Similarly, *KLK4* appears to be the product of a *KLK5* duplication which has occurred presumably after the marsupial-placental split (Figures 2 and 3). On the other hand, KLK10 and KLK12 appear to be sister groups (Figures 2 and 3), suggesting another duplication event. Since the Gly193Ser substitution is specific to KLK10 members (with the exception of platypus) it would be reasonable to suggest that this substitution took place after the *KLK10/KLK12* duplication. Interestingly, the branches of the KLK10 clade are exceptionally long, suggesting that the KLK10 members evolved more rapidly compared to the other KLKs.

The identification of an amphibian KLK1 permits to trace the evolutionary origin of KLKs 330 mya, when amphibia emerged [43]. However, our phylogenetic analysis showed that no proto-KLKs are present in the frog (Figures 2 and 3). One plausible explanation is that the ancestor of the reptilian orphan KLKs, a trypsin-like *proto-orphan KLK* emerged in amphibia; a series of gene duplication and deletion events gave rise to *KLK1-KLK15* that can be found in the contemporary genomes.

**Figure 6:**
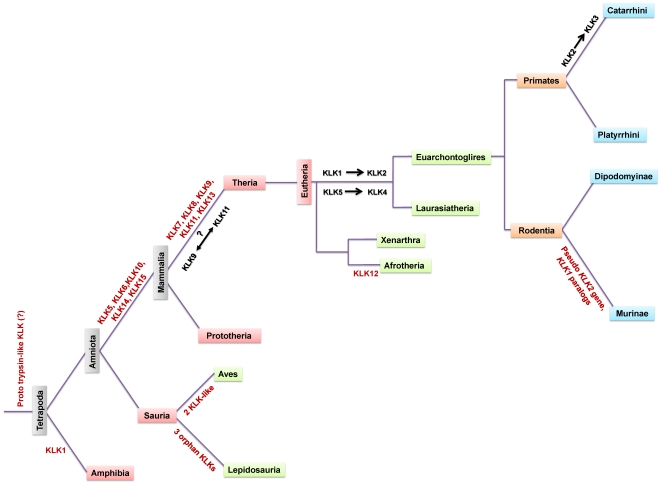
Evolution of KLKs. Schematic representation of key events occurring through the evolutionary history of KLKs.

The reconstructed phylogenetic tree in Figure S2 also demonstrates that the two piscine peptide sequences previously described as KLK-related [44] are totally unrelated to KLKs. Instead these proteins cluster with the known complement factor D/adipsin proteins [45], [46] (Figure S2). Finally, Figure 6 summarizes the evolution events in the KLK gene family.

### Rate shift analysis

**Figure 7:**
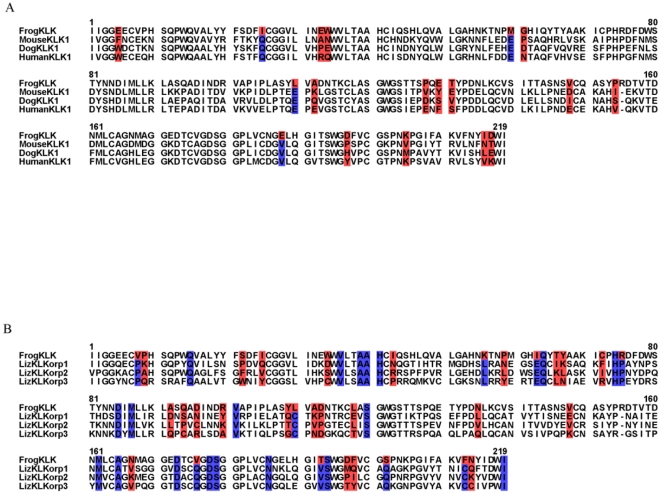
Rate shift analysis of frog KLK. **A, Comparison with orphan lizard KLKs and B, Comparison with KLK1.** Residues in blue and red background correspond to sites with slower and faster rate of evolution, respectively.

Rate shift analyses were carried out as described [47]. They were used to analyze the frog KLK and the subfamilies of KLK7, KLK10 and KLK2. Regarding the frog KLK, as shown in Figure 7 the most significant rate shifts occur between the frog KLK1 and the three orphan lizard KLKs (9 positions with significant rate shifts) rather between the frog and the other KLK1 proteins, where 4 positions with significant rate differences were found. These results support the phylogenetic analysis which suggests that the frog KLK is phylogenetically closer to the KLK1 proteins than to the lizard KLKs. For the KLK7, KLK10 and KLK12 it was found that lower rate shifts (12 positions) existed between KLK10 and KLK12 compared to KLK7 and KLK12 (16 positions) and KLK7 and KLK10 (26 positions) (Figure S3) which is also in accordance with the phylogenetic analysis results where the subfamilies KLK10 and KLK12 appear as sister groups.

### Genomic organization of the putative KLK-encoding genes

Inspection of the KLK protein sequences (Figure S1) suggests that they have virtually identical splicing patters, with slight deviations though. Several serine protease-encoding genes (toxin from Bushmaster, [48]) also have essentially the same splice sites with *KLKs*, where the three invariant catalytic residues are located on separate exons. This leads to the suggestion that the serine proteases evolved from an ancestral trypsin-like protein and have retained the same splicing patterns. Only, plasminogen (PLG)-encoding genes have different splicing patterns when compared to the rest of serine protease-encoding genes prompting that the split between the serine proteases took place at that time point [49].

## Discussion

The process of gene duplication is essential to the efficient generation of genes with novel or altered functions. When the duplicated gene is fixed to the genome and is functionally preserved by natural selection it may diverge either by neofunctionalization or subfunctionalization. The significance of this process is demonstrated by the widespread existence of gene families. The unique characteristic of co-localization and the large number of members is what makes the *KLK* gene family ideal for evolutionary studies. For example, *MMPs* constitute another important gene family (consisting of 25 genes in vertebrates and 24 in humans). MMPs are widely distributed in the animal kingdom and appear to have evolved from a single domain protein which underwent successive rounds of duplication, gene fusion and exon-shuffling. However, in contrast to *KLKs*, the members of this family are distributed along different chromosomes [50].

The increased availability of fully sequenced genomes from multiple organisms enabled us to conduct a detailed phylogenetic analysis of KLKs in order to reconstruct the evolutionary history of the KLK family. Contrary to the prevailing notion, in the present investigation it was shown that putative KLKs exist in non-therian species, covering an evolutionary distance from amphibia to eutheria. Previous work [28] suggested that no *KLK* genes were present in the genome of chicken, frog, or the song bird zebra finch [51]. However, our detailed analysis showed the existence of a frog (*Xenopus tropicalis*) *KLK* gene, confirmed the absence of a KLK-like sequence in chicken (*Gallus gallus*), but in contrast a KLK homologous sequence in turkey (*Meleagris gallopavo*) and a partial *KLK* gene (likely pseudogene) were revealed in zebra finch.

In view of our findings it would also be tempting to speculate that the evolutionary origin of KLKs should be moved further back to the radiation of amphibia (330 mya). Noticeably, despite extensive database searches no piscine, ascidian or insect KLK-related proteins were detected. The importance of our findings has implications for the physiological functions, while evolution of KLKs parallels that of their well-established substrates.

### Co-evolution of KLK enzymes and substrates

#### KLK2, KLK3 and reproductive physiology

*KLK2* and *KLK3* genes appeared later in evolution of the *KLK* gene family. Their functional roles are mainly linked to reproduction and more specifically to liquefaction of semen in humans [1], [5]. Of great interest is the fact that in gorilla the *KLK2* gene is absent (*i.e.* inactivated due to missing coding exons), as also absent are the KLK2-specific substrates in the seminal clot, *i.e.* semenogelin I, semenogelin II, and TGM4 (prostate transglutaminase 4) that are inactivated due to premature stop codons. Lack of seminal proteins diminishes the viscosity of semen that is liquefied upon ejaculation, therefore the KLK2 enzymatic activity is not needed in this case [40], [52]. We have further found that in *Macaca mullata* (Rhesus monkey), KLK2 has a mutation (active site Asp102Ala) that renders the enzyme inactive as previously reported [40]. This probably reflects differences in semen physiology between Rhesus monkey and humans, in that semen does not liquefy but instead forms a copulatory plug. Rhesus monkeys are polygamous in nature, therefore the presence of copulatory plug is important for sperm competition and mate guarding. On the contrary, gorillas are monogamous in nature and there is no need for mate guarding and sperm competition, therefore the aforementioned genes have been inactivated through selection. In addition, a copulatory plug does not exist in cow which is further characterized by the absence of *TGM4* [53], necessary for semen coagulum formation as well as absence of KLK2 and KLK3 that dissolve the coagulum. Further, absence of *TGM4* has been reported in opossum and again we did not find *KLK2* and *KLK3* genes in opossum [53]. Chimpanzee is another polygamous primate and although the genes encoding for KLK2 and KLK3 have not been deleted in this animal the gene for semenogelin I encodes for a more viscous protein of higher molecular weight compared to humans due to a greater number of repeated units [52]. Finally, in contrast to humans, in rodents, semen forms a hard rubbery plug upon ejaculation (copulatory plug). Rodents are highly polygamous in nature. The seminal vesicles of rats and mice secrete six proteins designated SVS1-6 from which SVS-2-6 are homologues to semenogelins [54], while semen also contains prostate transglutaminase [53]. These proteins cause plug formation, while absence of KLK2 and KLK3 prevents dissolution of the copulatory plug and, thus, rapid semen liquefaction.

#### KLK4 and tooth development

KLK4 is important for proteolysis and degradation of the 32 kDa fragment of enamelin since this procedure provides space for apatite growth. Retention of this fragment disturbs the biomineralization process. Consistently, knockout mice for *KLK4* showed abnormalities in teeth maturation [55] and humans suffering from autosomal recessive hypomaturation amelogenesis imperfecta carry a deactivating mutation in the KLK4 active site residue. Taken together this data indicate the crucial function of KLK4 in teeth development [56]. In *KLK4* knockout mice although the enamel layer thickness was normal it was rapidly abraded following weaning even when they were maintained with soft chow [55].

A very recent study reported that birds lack the enamelin-encoding gene which is in accordance with their lack of dentition [51]. One would expect that *KLK4* is unnecessary in these animals; indeed we were unable to detect this gene. Consistently, we showed here that chicken genome encodes a non-functional enamelin pseudogene and no KLK4 or other KLKs [57]. In the same context, the enamelin gene is present in monotremes [58], and while young animals have rudimentary teeth, adult monotremes lack dentition, and accordingly these animals are characterized by the absence of the *KLK4* gene as we could not detected it in platypus (Figure 4). Further, xenathra lack dentition, which renders a KLK4 enzyme unnecessary. Although indeed we could not detect *KLK4*, the presence of this gene in xenatha can not be definitively excluded due to incomplete contig information for armadillo. In contrast to the *KLK4* gene, enamelin gene is conserved in xenarthra [58].

#### KLKs and skin desquamation

It is well established that the skin desquamation process involves a proteolytic cascade, which is initiated by activation of proKLK5 either auto-catalytically [59] or by matriptase [60]. Subsequently, KLK5 activates proKLK7 and proKLK14. Mature KLK14 enhances proKLK5 activation in a feedback loop. In addition, it was shown recently that KLK5 is able to activate proelastase 2 *in vitro* indicating that KLK5 could be the physiological activator of proelastase 2 in epidermis [61]. Hyperactivation of KLKs (mainly KLK5 and KLK7) in epidermis has been implicated in pathological over-desquamation, a symptom common to a number of skin diseases, including atopic dermatitis and Netherton syndrome (NS) a rare syndrome of severe ichthyosis caused by mutations in *Spink5* gene that encodes LEKTI, a multidomain inhibitor of KLKs and other serine proteases [62]. *Spink5^−/−^* mice recapitulate the clinical phenotype of NS [62] as increased activities of KLK5, KLK7 and KLK14 due to lack of LEKTI result in enhanced proteolysis of their corneodesmosomal protein substrates (*i.e.* corneodesmosin, desmoglein and desmocollin) [63] that causes stratum corneum detachment and neonatal death. We found that corneodesmosin, desmoglein and desmocollin are present in platypus (ABU86923, XP_001515334 and XP_001515354, respectively) but in frogs only desmocollin was found (NP_001122136). This indicates that protein substrates that form the outer skin layer have co-evolved with their specific processing enzymes (*i.e*. KLK) as they are essential for replenishment of the skin surface. It is currently not clear whether the KLK skin cascade emerged in platypus, since we were unable to identify a KLK7 orthologue in platypus but this may be due to incomplete genome sequencing.

It should be noted that frog skin and the skin of amphibia, in general, is more permeable than that of mammals since it is engaged in respiration and regulation of internal water and ion loss [64]. For example, stratum corneum of frog epidermis is by 10 times thinner than that of pig [64]. Also, it should be noted that the stratum corneum originally appeared in amphibia and it was essential for terrestrial survival. Further, mouse skin is by 3 times less permeable than that of humans [65]. Interestingly, while human *SPINK5* encodes for LEKTI that contains 15 protease inhibitory domains, mouse and rat *Spink5* encode for LEKTI that contains only 14 domains and lacks domain 6 [66], the high-affinity inhibitor of KLK5 and 7 [63]. Therefore, it is expected that higher activity of KLK5 and KLK7 would be found in rodent skin compared to humans, which is compatible with its higher permeability due to increased desquamation. On the other hand, *Anolis carolinensis* (and generally lizards) has low-permeability skin. While putative orthologs for desmocollin (ENSACAG00000017830) and desmoglein (ENSACAG000000 17850) are encoded in lizards, the absence of KLK5 and KLK7 is compatible with decreased skin shedding and the low permeability of their stratum corneum. Additionally, skin permeability is also decreased by expression of hard-beta keratins and high amounts of lipids that “insulate” the skin [65].

### KLK1 loop-99 and adaptation to increased enzymatic activity

The loop-99 (starting at amino acid residue 99) is necessary for kininogenase activity and is present only in KLK1, KLK2 and PSA/KLK3 [1]. KLK1 is the prototypic kininogenase enzyme that cleaves low molecular-weight kininogen to release bradykinin. As shown in our analysis KLK1 appeared first in amphibia. Interestingly, Kita et al. [67] have reported the identification of a toxin in blarina, termed BLTX (blarina toxin), that displays high amino acid identity to human KLK1 (55.5 %). Recently, it was reported that amino acid substitutions and insertions mainly in the kallikrein loop are responsible for enhanced kininogenase activity that is expected to release increased amounts of bradykinin associated with toxicity [68]. We have determined in our phylogenetic analysis (data not shown) that Blarina toxin sorts into the KLK1 branch. Very recently, the presence in the platypus venom of an unknown enzyme with kininogenase activity was described [69]. It would be of particular interest, both from the physiological and evolutionary point of view, to determine the sequence of this enzyme and compare its structure with that of the KLK family members of platypus.

### Co-expression patterns of evolutionarily related KLKs and (patho)physiological functions

There is ample evidence that duplicated *KLK*s (i.e. *KLK2* and *KLK3*, *KLK4* and *KLK5*, *KLK*9 and *KLK11*, *KLK10* and *KLK12*) are coordinately regulated in biological fluids and tissues, while they often display common patterns of aberrant expression in disease states [1]. For example, *KLK*9 and *KLK11* are highly expressed in esophagus, vagina, stomach, breast, salivary gland and pancreas, *KLK4* and *KLK5* are highly co-expressed in breast and cervix, *KLK10* and *KLK12* in salivary gland, esophagus, fallopian tube, and pancreas [70], [71]. In this context, high levels of KLK5, 6, 7, 10, 12 and 13 have been detected in cervicovaginal fluid indicating potential role in cervical mucous remodeling and vagina epithelial desquamation [72], [73]. On the other hand, coordinated up-regulation of KLK5, 6, 7, 8, 10, 11 and 14 in ovarian cancer [74] and down-regulation of KLK5, 6, 8, and 10 in breast cancer [75] has been observed. *KLK* tissue-specific co-expression supports the hypothesis that each *KLK* gene is independently regulated by conserved regulatory mechanisms of transcription. Regulatory involvement of a locus control region (LCR) is not likely as the *KLK* locus evolved through a series of gene duplication events. Lack of a LCR is corroborated by studies showing that in transgenic mice bearing genomic fragment combinations of 2–3 neighboring rat *Klk* genes, rat-tissue *KLK* expression patterns are preserved [76].

Recent functional studies implicate certain KLKs in various types of cancer [1], [4], [7]. For example, in prostate cancer cells, expression of KLK3 and KLK4 results in loss of E-cadherin and induction of expression of the mesenchymal marker vimentin, a hallmark of epithelial-to-mesenchymal transitions, which is a critical step for cancer metastasis [77]. In contrast, re-expression of KLK6 at physiological concentrations dramatically inhibits the growth of primary breast tumors and causes marked reduction of vimentin [78]. Notably, KLK6 is known to be involved in demyelination by cleaving myelin basic protein [79] and to mediate E-cadherin shedding associated with wound healing *in vivo* [80]. Interestingly, certain KLKs may exert antiangiogenic functions, since they have been shown to release angiostatin-like peptides by proteolytic processing of plasminogen [81]. Recently, KLKs have emerged as versatile signaling molecules, since they were shown to act as activators of protease-activated receptors (PARs) [82] and the alpha(5)beta(1) integrin pathway [83].

### Conclusion

The fact that, during the course of evolution, KLKs have survived with significant similarity in terms of sequence, gene organization and number in higher organisms (from monotremes to primates) suggests that they likely play important roles in normal physiology. Elucidating the evolutionary history of KLKs would serve in the development of model systems for the study of gene function(s) in future studies. Collectively, the biological functions of the extended KLK family are currently under investigation. Pleiotropic physiological roles of KLK enzymes are being revealed, while aberrant regulation of KLKs is implicated in diverse diseases such as hypertension, renal dysfunction, skin disorders, inflammation, neurodegeneration, and cancer [4]. Experimental studies should be directed towards deciphering the biochemical function(s) of the putative KLK proteins.

## Methods

### Sequence database searching

In order to identify KLK orthologues, a combination of queries based on key terms and BLAST searches was employed. The names and/or accession numbers of the characterized kallikreins, including all human, mouse and rat KLKs, as well as the canine and equine prostate-specific antigen (KLK3), were used to retrieve their corresponding amino acid sequences. Then, the entire peptide sequences of those KLKs were used as probes to search the publicly available non-redundant databases, UniProt [84], GenBank [85] and Ensembl [86] applying reciprocal BLASTp and tBLASTn [87] (all E-values were below 1.0E-90). This process was reiterated until no novel sequences could be detected, ensuring that a full representation of the KLK family is obtained.

### Primary sequence analysis

The consensus boundaries of the core trypsin domain in the sequences included in the phylogenetic analyses, were determined from full-length sequences combining the outputs of Pfam [88], SMART [89], CD-Search [90], [91] and ScanProsite [92] protein domain prediction search engines. Moreover, using the FingerPRINTScan [93] search engine, a significant match to all three signature motifs held in PRINTS for the trypsin domain family was found. The sequences of these three conserved motifs for the human, opossum, platypus, lizard and frog KLK homologous proteins were used as input to Weblogo [94] to produce a consensus sequence for the three KLK catalytic motifs.

### Secondary structure prediction

The secondary structure of the identified putative KLK homologous proteins was predicted as a consensus (*i.e.* 3 out of 5 predictions) of the combined output of CDM [95], Jpred3 [96], Porter [97], PSIPRED [98] and SSpro [99]. The novel KLK amino acid sequences were aligned along with three KLKs with resolved tree-dimensional structure using PROMALS3D [100], [101], a multiple sequence alignment program which incorporates structural information in order to improve alignment accuracy.

### Tertiary structure analysis

The program ConSurf [102] was employed to estimate the degree of conservation of amino acid residues of putative KLKs. For this purpose, the multiple sequence alignment output of the entire KLK homologous amino acid sequences analyzed in this study was used as input to the program to project the conservation grades of residues on the known three-dimensional structure of the human proKLK6 (PDB ID: 1gvl) [34]. For molecular modeling the PyMol molecular graphics program was used.

### Phylogenetic analysis

The predicted core trypsin domain was excised from the full-length peptide sequence analyzed here. The cropped sequences were subsequently aligned using MUSCLE [103] and phylogenetic trees were reconstructed by employing PhyML [104], [105], a maximum likelihood (ML)-based program which optimizes a seed Neighbor-Joining tree by using a simple hillclimbing algorithm. The LG [106] amino acid substitution model was used. Bootstrap analysis (500 replicates) was performed to test the robustness of the inferred trees. The resulting phylogenetic trees were visualized with Dendroscope [107].

### Chromosomal positioning of KLK genes

The chromosomal localization of the genes encoding for the KLK homologous proteins was identified using the Ensembl GeneView [86] and NCBI MapViewer [108].

### Genomic organization of putative KLK homologues

The genomic organization of putative *KLK*-encoding genes was analyzed by identifying the boundaries between the exons encoding the core trypsin domain of the KLK homologous proteins. The exon-intron boundaries were identified in ENSEMBL. The splice sites were also verified using the core domain of the amino acid sequences shown in Figure S1 as seeds in a tBLASTn search against their respective genomes. The consecutive encoding exons were retrieved in this way. The splicing patterns of several other genes coding for serine proteases such as trypsins, chymotrypsins, CFD, and plasminogens (PLG) [49] and KLK-like toxin [48] were also analyzed.

### Rate shift analysis

Rate shift analyses were carried out using the program available at: http://www.daimi.au.dk/~compbio/rateshift [47].

## Supporting Information

Figure S1 Exon-exon structure of KLKs. Multiple alignment of the amino acid sequences corresponding to the core trypsin domain of KLKs and other serine proteases. The sequences were aligned using MUSCLE. The numbers refer to the amino acid positions with respect to the starting position of the core domain. The spice sites are denoted at the beginning of the respective exons as white letters in a black background. The exon boundaries of particular note are shown in a magenta background. The three catalytic triad residues are shown in blue and the glycine residue in green.
(0.02 MB PDF)

Figure S2 ML phylogram of KLK homologues and related proteins. The CFD/Adipsin sequences were included in the phylogenetic analysis as well. The sequences which are subject to question are indicated by arrows. Conventions are the same as in Figure 7.
(0.26 MB TIF)

Figure S3 Rate shift analysis of KLK7, 10, and 12 subfamilies. The analysis further supports our phylogenetic analysis by demonstrating that KLK10 and KLK12 subfamilies are sister groups.
(5.08 MB TIF)

Table S1 Names and accession numbers of the sequences analyzed in the present study. The KLK pseudogene names are shown in italics.
(0.32 MB DOC)
