# Supplementary material for: Evolutionary History of Tissue Kallikreins
Source: PLoS One. 2010 Nov 1;5(11):e13781. doi: 10.1371/journal.pone.0013781 (PMC2967472; doi:10.1371/journal.pone.0013781)
Supplement: Figure S1 — Exon-exon structure of KLKs. Multiple alignment of the amino acid sequences corresponding to the core trypsin domain of KLKs and other serine proteases. The sequences were aligned using MUSCLE. The numbers refer to the amino acid positions with respect to the starting position of the core domain. The spice sites are denoted at the beginning of the respective exons as white letters in a black background. The exon boundaries of particular note are shown in a magenta background. The three catalytic triad residues are shown in blue and the glycine residue in green. (0.02 MB PDF) [file pone.0013781.s001.pdf]

```
Human KLK1              IVGGWECEQHSQPWQAALYHF--ST----FQCGGILVHRQWVLTAAHCI----SD-NYQLWLGRHNLFDD-E-------NTAQFVHVSES 71
Frog KLK1               IIGGEECVPHSQPWQVALYYF--SD----FICGGVLINEWWVLTAAHCI----QS-HLQVALGAHNKTNP-M-------GHIQYTYAAKI 71
Human KLK2              IVGGWECEKHSQPWQVAVYSH--GW----AHCGGVLVHPQWVLTAAHCL----KK-NSQVWLGRHNLFEP-E-------DTGQRVPVSHS 71
Human KLK3              IVGGWECEKHSQPWQVLVASR--GR----AVCGGVLVHPQWVLTAAHCI----RN-KSVILLGRHSLFHP-E-------DTGQVFQVSHS 71
Human KLK4              IINGEDCSPHSQPWQAALVME--NE----LFCSGVLVHPQWVLSAAHCF----QN-SYTIGLGLHSLEADQE-------PGSQMVEASLS 72
Human KLK5              IINGSDCDMHTQPWQAALLLRP-NQ----LYCGAVLVHPQWLLTAAHCR----KK-VFRVRLGHYSLSPVYE-------SGQQMFQGVKS 73
Opossum KLK5            ILNGKDCQPQTQPWQAALFLRP-NR----LYCGAVLVHPQWVLTAAHCQ----KP-AYSVRLGRYHLQGI-D-------PGQRILRGVKS 72
Human KLK6              LVHGGPCDKTSHPYQAALYTS--GH----LLCGGVLIHPLWVLTAAHCK----KP-NLQVFLGKHNLRQR-E-------SSQEQSSVVRA 71
Platypus KLK6           IIAGEKCKEDGHPYQVAVYRG--GG----LLCGGVLIHPGWVLTAAHCR----KP-QLQVLLGKYNLHKQ-E-------EFQQLSPVAQP 71
Human KLK7              IIDGAPCARGSHPWQVALLSG--NQ----LHCGGVLVNERWVLTAAHCK----MN-EYTVHLGSDTLG---D-------RRAQRIKASKS 69
Opossum KLK7            IIQGVECNKNTHPWQVALFDG--NQ----LHCGGVLVAANWVLTAAHCR----KG-SYNVHLGKHSLKGN-E-------KGSQVIRATRS 71
Human KLK8              VLGGHECQPHSQPWQAALFQG--QQ----LLCGGVLVGGNWVLTAAHCK----KP-KYTVRLGDHSLQNK-D-------GPEQEIPVVQS 71
Human KLK9              AIGAEECRPNSQPWQAGLFHL--TR----LFCGATLISDRWLLTAAHCR----KP-YLWVRLGEHHLWKW-E-------GPEQLFRVTDF 71
Opossum KLK9            AIGAEECTPNSQPWQAGLFFL--TR----LFCGATLLNDQWLLTAAHCR----KP-YLWVRLGEHHLWKW-E-------GPEQLFRVTDF 71
Human KLK10             EAYGAPCARGSQPWQVSLFNG--LS----FHCAGVLVDQSWVLTAAHCG----NK-PLWARVGDDHLLLL---------QGEQLRRTTRS 70
Platypus KLK10          ------CGRDSHPWQVSLFRN--LK----FRCAGVLVDRSWVLTSATCI----DR-SWPVRPRGKGQQGT-L-------LHAKLTVQMNK 65
Human KLK11             IIKGFECKPHSQPWQAALFEK--TR----LLCGATLIAPRWLLTAAHCL----KP-RYIVHLGQHNLQKE-E-------GCEQTRTATES 71
Opossum KLK11           IIKGYECPAHSQPWQVALFQK--SR----LHCGATLINRQWLLTAAHCK----KP-QYWVYLGEHNLQRP-D-------SCEQKRMATLS 71
Human KLK12             IFNGTECGRNSQPWQVGLFEG--TS----LRCGGVLIDHRWVLTAAHCS----GS-RYWVRLGEHSLSQL-D-------WTEQIRHSGFS 71
Human KLK13             LPGGYTCFPHSQPWQAALLVQ--GR----LLCGGVLVHPKWVLTAAHCL----KE-GLKVYLGKHALGRV-E-------AGEQVREVVHS 71
Opossum KLK13           LPGVKECIPHSQPWQAVLLVN--GR----LLCGGILIHPSWVLTAAHCL----KN-KYTVYLGKHALGYS-E-------AGEQARRVTCS 71
Human KLK14             IIGGHTCTRSSQPWQAALLAGPRRR----FLCGGALLSGQWVITAAHCG----RP-ILQVALGKHNLRRW-E-------ATQQVLRVVRQ 73
Opossum KLK14           IIGGYTCIPHSQPWQAALFAS--RK----FHCGGVLLSNRWVLTAAHCG----HW-NLRVALGKHNLQRL-E-------PSQQMLRVNRQ 71
Platypus KLK14          ------------------------------------------------------R-FLRVSLGKHDLRWW-E-------PTEQRRLVVRQ 27
Human KLK15             LLEGDECAPHSQPWQVALYER--GR----FNCGASLISPHWVLSAAHCQ----SR-FMRVRLGEHNLRKR-D-------GPEQLRTTSRV 71
Opossum KLK15           VLGGEECPPHSQPWQVALFEG--RR----FNCGASLISSQWLLTAAHCY----TS-KMRVRLGEHNLRVW-D-------GMEQLRGVVYA 71
Platypus KLK15          VLGGKECAPGSQPWQVALFDF--GR----FNCGGTLLTPRWVLTAAHCL----TR-PIRVRLGEHNLKVL-E-------GQEQLRGVSRA 71
Lizard KLK orphan 1     VPGGKACPAHSQPWQAGLFSG--FR----LVCGGTLIHKSWVLSAAHCR----RRSPFPVRLGEHDLKRL-D-------WSEQLKLASKV 72
Lizard KLK orphan 2     IIGGYNCPQRSRAFQAALVTG--RRGNWNIYCGGSLVHPCWVLSAAHCK----PRRQMKVCLGKSNLRRY-E-------RTEQCLNIAEV 76
Lizard KLK orphan 3     IIGGQECPKHGQPYQVILSNS--RKNGPDVQCGGVLIDKDWVLTAAHCD----NQGTIHTRMGDHSLRAN-E-------GSEQCIKSAQK 76
Lizard KLKL toxin       IIGGEECAEDGHPWLVSIYGS--NW----LQCSGVLINQDWVLTAAHCY----KRSGMQLKLGVHNKEVP-R-------EGEQLRTSAEA 72
Human Trypsin          IVGGYTCEENSLPYQVSLNS---GS----HFCGGSLISEQWVVSAAHCY----KT-RIQVRLGEHNIKVL-E-------GNEQFINAAKI 70
Frog Trypsin           IIGGSTCARNSVPYIVSLNA---GY----HFCGGSLLNNQWVVSAAHCY----QA-SIQVRLGEHNIALN-E-------GTEQFINSAKV 70
Zebrafish Trypsin      IVGGYECTKNGVPYQVSLNS---GY----HFCGGSLISNLWVVSAAHCY----KS-RVQVRLGEHNIDVT-E-------GTEQFINSEKV 70
Human Chymotrypsin     VVGGEDARPHSWPWQISLQYL--KDDTWRHTCGGTLIASNFVLTAAHCI---SNTWTYRVAVGKNNLEVEDE-------EGSLFVGVDTI 78
Zebrafish Chymotrypsin IVNGEEARPHSWPWQVSLQDST-GF----HFCGGSLINENWVVTAAHCN----VRTSHRVILGEHDRSSN-A-------EAIQTIAVGKS 73
Human CFD              ILGGREAEAHARPYMASVQLN--GA----HLCGGVLVAEQWVLSAAHCLEDAADG-KVQVLLGAHSLSQP-E-------PSKRLYDVLRA 75
Zebrafish CFD          ITGGQEAKAHSRPYMASVQWN--GK----HECGGFLISSQWVMSAAHCFQDGRTS-GVKVVLGAHSLSGA-E-------DTKQTFD-AEV 74
Human PLG              VVGGCVAHPHSWPWQVSLRTRF-GM----HFCGGTLISPEWVLTAAHCLEKSPRPSSYKVILGAHQEVNL-E-------PHVQEIEVSRL 77
Zebrafish PLG          IVGGCVSKPHSWPWQISLRTR--GK---IHFCGGTLIDPQWVVTAAHCLERSDSPSAYKIMLGIHTERAT-E-------SSKQERDVTKI 77
```

```
Human KLK1             FPHPGFNMSLLENHTRQADEDYSHDLMLLRLTEPADTITDAVKVVELP-TQE--PEV-GSTCLASGWGSIEP----------ENF----- 142
Frog KLK1              CPHRDF--------DWS---TYNNDIMLLKLASQAD-INDRVAPIPLA-SYL--VAD-NTKCLASGWGSTTS----------PQE----- 131
Human KLK2             FPHPLYNMSLLKHQSLRPDEDSSHDLMLLRLSEPAK-ITDVVKVLGLP-TQE--PAL-GTTCYASGWGSIEP----------EEF----- 141
Human KLK3             FPHPLYDMSLLKNRFLRPGDDSSHDLMLLRLSEPAE-LTDAVKVMDLP-TQE--PAL-GTTCYASGWGSIEP----------EEF----- 141
Human KLK4             VRHPEY--------NRP---LLANDLMLIKLDESVS-ESDTIRSISIA-SQC--PTA-GNSCLVSGWGLLAN------------G----- 130
Human KLK5             IPHPGY--------SHP---GHSNDLMLIKLNRRIR-PTKDVRPINVS-SHC--PSA-GTKCLVSGWGTTKS----------PQV----- 131
Opossum KLK5           FPHPKY--------TRP---AHSNDLMLIKLNGRVS-MTPSIKTIKIS-TQP--PRP-GTQCLVSGWGTTSS----------PQV----- 131
Human KLK6             VIHPDY--------DAA---SHDQDIMLLRLARPAK-LSELIQPLPLE-RDC--SAN-TTSCHILGWGKTAD------------G----- 128
Platypus KLK6          FPHPDY--------DRG---RHDNDIMLLRLAHPTP-VSRHIKPLSLE-TDC--NAN-SSSCLISGWVFVKH----------SDG----- 128
Human KLK7             FRHPGY--------STQ---THVNDLMLVKLNSQAR-LSSMVKKVRLP-SRC--EPP-GTTCTVSGWGTTTS----------PDV----- 130
Opossum KLK7           YRHPQY--------STV---THANDFLLIRLSRPAR-IGPTIKPITLP-SRC--APP-GTSCTVSGWGTTTS----------PDV----- 133
Human KLK8             IPHPCYNSS-----DVE---DHNHDLMLLQLRDQAS-LGSKVKPISLA-DHC--TQP-GQKCTVSGWGTVTS----------PRE----- 134
Human KLK9             FPHPGFNKDL----SAN---DHNDDIMLIRLPRQAR-LSPAVQPLNLS-QTC--VSP-GMQCLISGWGAVSS----------PKA----- 134
Opossum KLK9           FPHPGFNSDL----SAH---DHNHDIMLIRLKRPAK-LGPAVQPLNIS-NNC--VRP-GTTCLISGWGATSS----------PEV----- 137
Human KLK10            VVHPKYHQGSGPILPRR---TDEHDLMLLKLARPVV-PGPRVRALQLP-YRC--AQP-GDQCQVAGWGTTAA----------RRV----- 132
Platypus KLK10         YHRPGERCGTWTAGAGF----GEQGLGRVWKAGRGC-WRVGMDPGGWV-WGL--RRG-GSTCKV-GVGPEGE----------STV----- 132
Human KLK11            FPHPGFNNSL----PNK---DHRNDIMLVKMASPVS-ITWAVRPLTLS-SRC--VTA-GTSCLISGWGSTSS----------PQL----- 134
Opossum KLK11          FPHPGFNGSL----PNK---DHRNDIMLVKMNSPVM-LTRGVKPLALP-EDC--ATA-GSQCLISGWGTTTS----------PQL----- 132
Human KLK12            VTHPGYLG------AST---SHEHDLRLLRLRLPVR-VTSSVQPLPLP-NDC--ATA-GTECHVSGWGITNH----------PRN----- 134
Human KLK13            IPHPEYRRSP----THL---NHDHDIMLLELQSPVQ-LTGYIQTLPLSHNNR--LTP-GTTCRVSGWGTTTS----------PQV----- 135
Opossum KLK13          VPHPLYRGIP----THQ---SHDHDIMLLHLHKPAQ-LNNQVQVLPLPSQEY--LPP-GTCCTVSGWGTTTS----------PQV----- 135
Human KLK14            VTHPNY--------NSR---THDNDLMLLQLQQPAR-IGRAVRPIEVT-QAC--ASP-GTSCRVSGWGTISS----------PIA----- 130
Opossum KLK14          VVYPRY--------NQR---TNDHDLMLLHLERPVK-LTREIQPIQVA-RNC--ASP-GTSCLVSGWGTISS----------PHV----- 86
Platypus KLK14         IPHPSY--------NPR---TQDSDLMLLRLDRPVQ-LTPAVQPLGLS-PDC--AAP-GTPCRVSGWGTISS----------PLASPGPS 135
Human KLK15            IPHPRY--------EAR---SHRNDIMLLRLVQPAR-LNPQVRPAVLP-TRC--PHP-GEACVV-GWGLVSHNEPGTAGSPRSQV----- 139
Opossum KLK15          FPHPRY--------QGH---GHHDDIMLLRLARPAS-LSDSVQPVALP-VRC--PRT-GEQCVVSGWGLVAESNNKTQRAPVPKV----- 140
Platypus KLK15         IPHPRY--------RER---DHEDDIMLLKLALPAR-LTPSVRPLSLP-TRC--PQP-GDRCVVSGWGLVAN----------SK------ 130
Lizard KLK orphan 1    IVHPNY--------DPQ---TKNNDIMLVKLLTPVC-LNNKVKILKLP-TTC--PVP-GTECLISGWGTTTS----------PEV----- 135
Lizard KLK orphan 2    RVHPEY--------DRS---KNNKDYMLLRLQPCAR-LSDAVKTIQLP-SGC--PND-GKQCTVSGWGTTRS----------PQA----- 135
Lizard KLK orphan 3    FIHPAY--------NPS---THDSDIMLIRLDNSAN-INEYVRPIELA-TQC--TKP-NTRCEVSGWGTIKT----------PQS----- 139
Lizard KLKL toxin     KCYPDAASSDSNNCAEK---TFVNDIMLVKLNAPVD-YNDNIQPVPLP-TAV--PEV-GTECKVMGWGTIHS----------SED----- 129
Human Trypsin          IRHPKY--------NRD---TLDNDIMLIKLSSPAV-INARVSTISLP-TAP--PAA-GTECLISGWGNTLS----------FGA----- 129
Frog Trypsin           IRHPNY--------NSR---TIDNDIMLIKLASPAS-LNSNVNAVALP-SSC--AAA-GSSCLISGWGNTST----------SGS----- 129
Zebrafish Trypsin      IRHPSY--------NSN---TLDNDVMLIKLSSSAQ-INSYVKTVSLP-SSC--ASS-GTSCLISGWGNMSA----------SGS----- 130
Human Chymotrypsin     HVHKRW--------NAL---LLRNDIALIKLAEHVE-LSDTIQVACLPEKDS--LLPKDYPCYVTGWGRLWT----------NG------ 133
Zebrafish Chymotrypsin IKHPNY--------NSF---TINNDILLIKLATPAK-INTHVSPVCLAETND--NFPGGMKCVTSGWGLTRY----------NAP----- 136
Human CFD              VPHPDS--------QPD---TIDHDLLLLQLSEKAT-LGPAVRPLPWQRVDR-DVAP-GTLCDVAGWGIVNH----------AG------ 134
Zebrafish CFD          YNHPDF--------SIS---NYDNDIALIKLDKPVT-QSDAVKPVKFQRDETADPKE-AAVVETAGWGSLNN----------MG------ 140
Human PLG              FLEPTR-----------------KDIALLKLSSPAV-ITDKVIPACLPSPNY--VVADRTECFITGWGETQG----------T------- 130
Zebrafish PLG          IKGPAG-----------------TDIALLKLDRPAL-INDKVSPVCLPEKDY--IVPSNTECYVTGWGETQD----------T------- 139
```

```
Human KLK1             ----SFPDDLQCVDLKILPNDECEK-AHV--Q---KVTDFMLCVGHLEGGKDTCV-GDSGGPLMC---DG----VLQ-GVTSWGYV-PCG 201
Frog KLK1              ----TYPDNLKCVSITTASNSVCQA-SYPR-D---TVTDNMLCAGNMAGGEDTCV-GDSGGPLVC---NG----ELH-GITSWGDF-VCG 212
Human KLK2             ----LRPRSLQCVSLHLLSNDMCAR-AYS--E---KVTEFMLCAGLWTGGKDTCG-GDSGGPLVC---NG----VLQ-GITSWGPE-PCA 211
Human KLK3             ----LTPKKLQCVDLHVISNDVCAQ-VHP--Q---KVTKFMLCAGRWTGGKSTCS-GDSGGPLVC---NG----VLQ-GITSWGSE-PCA 201
Human KLK4             ----RMPTVLQCVNVSVVSEEVCSK-LYD--P---LYHPSMFCAGGGQDQKDSCN-GDSGGPLIC---NG----YLQ-GLVSFGKA-PCG 200
Human KLK5             ----HFPKVLQCLNISVLSQKRCED-AYP--R---QIDDTMFCAGDKA-GRDSCQ-GDSGGPVVC---NG----SLQ-GLVSWGDY-PCA 200
Opossum KLK5           ----HYPQVLQCLNITIMSHEACQR-AYP--G---AIDSTMFCAGDEV-GKDSCQ-GDSGGPVVC---NG----YLQ-GLVSWGDV-PCG 199
Human KLK6             ----DFPDTIQCAYIHLVSREECEH-AYP--G---QITQNMLCAGDEKYGKDSCQ-GDSGGPLVC---GD----HLR-GLVSWGNI-PCG 198
Platypus KLK6          ----GGAREGRSERVSPPDNTECYLHVHPKQREPSRAADAIVEGGESGRGLGKAQKGDSGGPLVC---GG----RLR-GLVSWGEV-PCG 205
Human KLK7             ----TFPSDLMCVDVKLISPQDCTK-VYK--D---LLENSMLCAGIPDSKKNACN-GDSGGPLVC---RG----TLQ-GLVSWGTF-PCG 200
Opossum KLK7           ----TYPDKLQCTDVKLISFSECKK-VYK--D---LLKESMLCAGIPGSSTNACN-GDSGGPLVC---NG----VLE-GLVSWGTF-PCG 203
Human KLK8             ----NFPDTLNCAEVKIFPQKKCED-AYP--G---QITDGMVCAGSSK-GADTCQ-GDSGGPLVC---DG----ALQ-GITSWGSD-PCG 203
Human KLK9             ----LFPVTLQCANISILENKLCHW-AYP--G---HISDSMLCAGLWEGGRGSCQ-GDSGGPLVC---NG----TLA-GVVSGGAE-PCS 204
Opossum KLK9           ----EYPLSLQCANISVLDPRLCHK-AYP--G---RITSNMVCAGLWEGGRGSCQ-GDSGGPLVC---NG----ALA-GVVSGGAE-PCS 207
Human KLK10            ----KYNKGLTCSSITILSPKECEV-FYP--G---VVTNNMICAGLDR-GQDPCQ-SDSGGPLVC---DE----TLQ-GILSWGVY-PCG 201
Platypus KLK10         ----KYAKNLSCAPVKVLSHEECSQ-SYP--G---VVTRNMLCAG-QAGGRDPCQ-GDSGGPLVC---NG----TLQ-GILSWGDY-PCG 201
Human KLK11            ----RLPHTLRCANITIIEHQKCEN-AYP--G---NITDTMVCASVQEGGKDSCQ-GDSGGPLVC---NQ----SLQ-GIISWGQD-PCA 204
Opossum KLK11          ----TLPHTLRCANITLIDHRECEG-AYP--G---NITDTMVCAGVTKEGKDSCQ-GDSGGPLVC---NG----TLQ-GIISWGQD-PCA 202
Human KLK12            ----PFPDLLQCLNLSIVSHATCHG-VYP--G---RITSNMVCAGGVP-GQDACQ-GDSGGPLVC---GG----VLQ-GLVSWGSVGPCG 204
Human KLK13            ----NYPKTLQCANIQLRSDEECRQ-VYP--G---KITDNMLCAGTKEGGKDSCE-GDSGGPLVC---NR----TLY-GIVSWGDF-PCG 205
Opossum KLK13          ----NYPKTLQCAEIQLRSDEECHQ-SYP--G---KITPNMLCAGSQEGGKDSCE-GDSGGPLVC---NG----ILQ-GVISWGDF-PCG 203
Human KLK14            ----RYPASLQCVNINISPDEVCQK-AYP--R---TITPGMVCAGVPQGGKDSCQ-GDSGGPLVC---RG----QLQ-GLVSWGME-RCA 200
Opossum KLK14          ----RYPSTLQCVNIDILSDERCRN-SYP--G---AITRGMVCAGDQKGEKDSCQ-GDSGGPLVC---NG----ALQ-GLVSWGSE-QCA 156
Platypus KLK14         PSPVRYPDILQCVNIQIVSDKACQG-AYP--G---GITPSMVCAGDWQGGKDSCQ-GDSGGPLVC---RG----KLQ-GLVSWGLE-QCG 209
Human KLK15            ----SLPDTLHCANISIISDTSCDK-SYP--G---RLTNTMVCAGAEGRGAESCE-GDSGGPLVC---GG----ILQ-GIVSWGDV-PCD 209
Opossum KLK15          ----KLPDTLHCANISIISAASCNR-DYP--G---QVTDTMVCAGVEGGGTDSCE-GDSGGPLVC---GG----TLQ-GIVSWGDV-PCD 210
Platypus KLK15         -------------------------------------------GEEAGGTDRWV-GC------------------------------- 143
Lizard KLK orphan 1    ----NFPDVLHCANITVVDYEVCRS-IYP--N---YINENMVCAGKMEGGTDACQ-GDSGGPLAC---NG----QLQ-GIVSWGPI-LCG 205
Lizard KLK orphan 2    ----QLPAQLQCANVSIVPQPKCNS-AYR--G---SITPYMVCAGVPQGGTDSCQ-GDSGGPLVC---NG----QLE-GVVSWGTY-VCA 205
Lizard KLK orphan 3    ----EFPDLLQCATVYTISNEECNK-AYP--N---AITENMLCATVSGGGVDSCQ-GDSGGPLVC---NN----KLQ-GIVSWGMQ-VCA 209
Lizard KLKL toxin      ----IYPLVPYCVDIEILNNGVCEA-AYPW-C---TAYDKLLCAGVLEGGKDSCH-GDSGGPLMC---GD----ELQ-GILSFGGH-PCA 200
Human Trypsin          ----DYPDELKCLDAPVLTQAECKA-SYP--G---KITNSMFCVGFLEGGKDSCQ-RDSGGPVVC---NG----QLQ-GVVSWGH--GCA 198
Frog Trypsin           ----NYPNLLQCLSAPILTTAQCTG-AYP--G---QITNNMFCAGFLEGGKDSCQ-GDSGGPVVC---NG----QLQ-GIVSWGV--GCA 198
Zebrafish Trypsin      ----NYPSRLMCLNAPILSDSTCRN-AYP--G---QISSNMFCAGFMEGGKDSCQ-GDSGGPVVC---NN----QLQ-GIVSWGY--GCA 199
Human Chymotrypsin     ----PIADKLQQGLQPVVDHATCSRIDWW--GF--RVKKTMVCAGG-DGVISACN-GDSGGPLNCQLENG--SWEVF-GIVSFGSRRGCN 210
Zebrafish Chymotrypsin ----DTPALLQQAALPLLTNDDCKR--YW--GT--NITDLMICAGAS--GVSSCM-GDSGGPLVC---ENNRVWTLV-GIVSWGSS-TCS 208
Human CFD              ----RRPDSLQHVLLPVLDRATCNRRTHHD-G---AITERLMCAESNR--RDSCK-GDSGGPLVC---GG----VLE-GVVTSGSR-VCG 204
Zebrafish CFD          ----GRPDKLHELSIPVMERWRCGRADFY--GE--KFTSNMLCAADKR--KDTCD-GDSGGPLLY---RG----IVV-GITSNGGK-KCG 210
Human PLG              ----FGAGLLKEAQLPVIENKVCNRYEFLN-G---RVQSTELCAGHLAGGTDSCQ-GDSGGPLVC---FEKDKYILQ-GVTSWGL--GCA 205
Zebrafish PLG          ----GGEGYLKETGFPVIENKVCNRPSFLN-G---RVKDHEMCAGNIEGGNDSCQ-GDSGGPLVC---YAQNTFVLQ-GVTSWGL--GCA 214
```

```
Human KLK1             TP------------------NKPSVAVRVLSYVKWIEDTIA 235
Frog KLK1              SP------------------NKPGIFAKVFNYIDWINNIMQ 224
Human KLK2             LP------------------EKPAVYTKVVHYRKWIKDTIA 234
Human KLK3             LP------------------ERPSLYTKVVHYRKWIKDTIV 234
Human KLK4             QV------------------GVPGVYTNLCKFTEWIEKTVQ 222
Human KLK5             RP------------------NRPGVYTNLCKFTKWIQETIQ 224
Opossum KLK5           QP------------------NKPGVYTNLYVFEKWIEDTIK 223
Human KLK6             SK------------------EKPGVYTNVCRYTNWIQKTIQ 221
Platypus KLK6          TG------------------EKPGVYTNVCRYVDWIRETIR 230
Human KLK7             QP------------------NDPGVYTQVCKFTKWINDTMK 221
Opossum KLK7           RS------------------NDPGVYSQVCKQMPWILSVIR 223
Human KLL8             RP------------------DKPGVYTNICRYLDWIKKIIG 225
Human KLK9             KP------------------RRPAVYTSVCHYLDWIQEIME 227
Opossum KLK9           SA------------------KRPSVYTSVCQYRKWISKTMR 227
Human KLK10            AG------------------QHPAVYTQICKYMSWINKVIR 229
Platypus KLK10         IT------------------PHPAVYTKICRYSSWINKIIK 222
Human KLK11            VS------------------RKPGVYTKVCKYVDWIQETMK 227
Opossum KLK11          QD------------------RKPGVYTKVCKYVDWIHETIK 227
Human KLK12            QP------------------GIPGVYTYICKYVDWIRMIMR 225
Human KLK13            QP------------------DRPGVYTRVSRYVLWIRETIR 228
Opossum KLK13          LP------------------NRPGVYTRVSRYVDWIQNVIQ 228
Huma KLK14             QA------------------GYPGVYTNLCKYRSWIEETMR 225
Opossum KLK14          LS------------------KYPGVYTNLCRYWTWIQNEVG 223
Platypus KLK14         NT------------------NHPGVYTNLCKFTRWIQNTMR 188
Human KLK15            TT------------------TKPGVYTKVCHYLEWIRETMK 232
Opossum KLK15          TT------------------TKPGVYTKVCKYLTWIRDTMK 233
Platypus KLK15         ----------------------------------------- 143
Lizard KLK orphan 1    QP------------------NRPGVYVNVCKYVDWIRETIR 224
Lizard KLK orphan 2    QK------------------GNPGVYAKVCCIVPWIQKTVN 228
Lizard KLK orphan 3    QA------------------GKPGVYTNICQFTDWIQNTMR 228
Lizard KLKL toxin      KPLEPGVYVKVDSYLDWITTTKPGVYTKVCKYLTWIRDTMK 251
Human Trypsin          WK------------------NRPGVYTKVYNYVDWIKDTIA 221
Frog Trypsin           QR------------------NYPGVYTKVCNYNSWIQSTIA 221
Zebrafish Trypsin      QR------------------NKPGVYAKVCTSPPGSETP-- 219
Human Chymotrypsin     TR------------------KKPVVYTRVSAYIDWINEKMQ 238
Zebrafish Chymotrypsin T-------------------STPAVYARVTKLRAWVDQTIA 228
Human CFD              NR------------------KKPGIYTRVASYAAWIDSVLA 228
Zebrafish CFD          SS------------------RKPGLYTIISHYASWIDTTTT 228
Human PLG              RP------------------NKPGVYVRVSRFVTWIEGVMR 228
Zebrafish PLG          NA------------------MKPGVYTRVSKFVDWIERSIK 228
```
