# Supplementary figures and images for: Evolutionary History of Tissue Kallikreins
Source: PLoS One. 2010 Nov 1;5(11):e13781. doi: 10.1371/journal.pone.0013781 (PMC2967472; doi:10.1371/journal.pone.0013781)

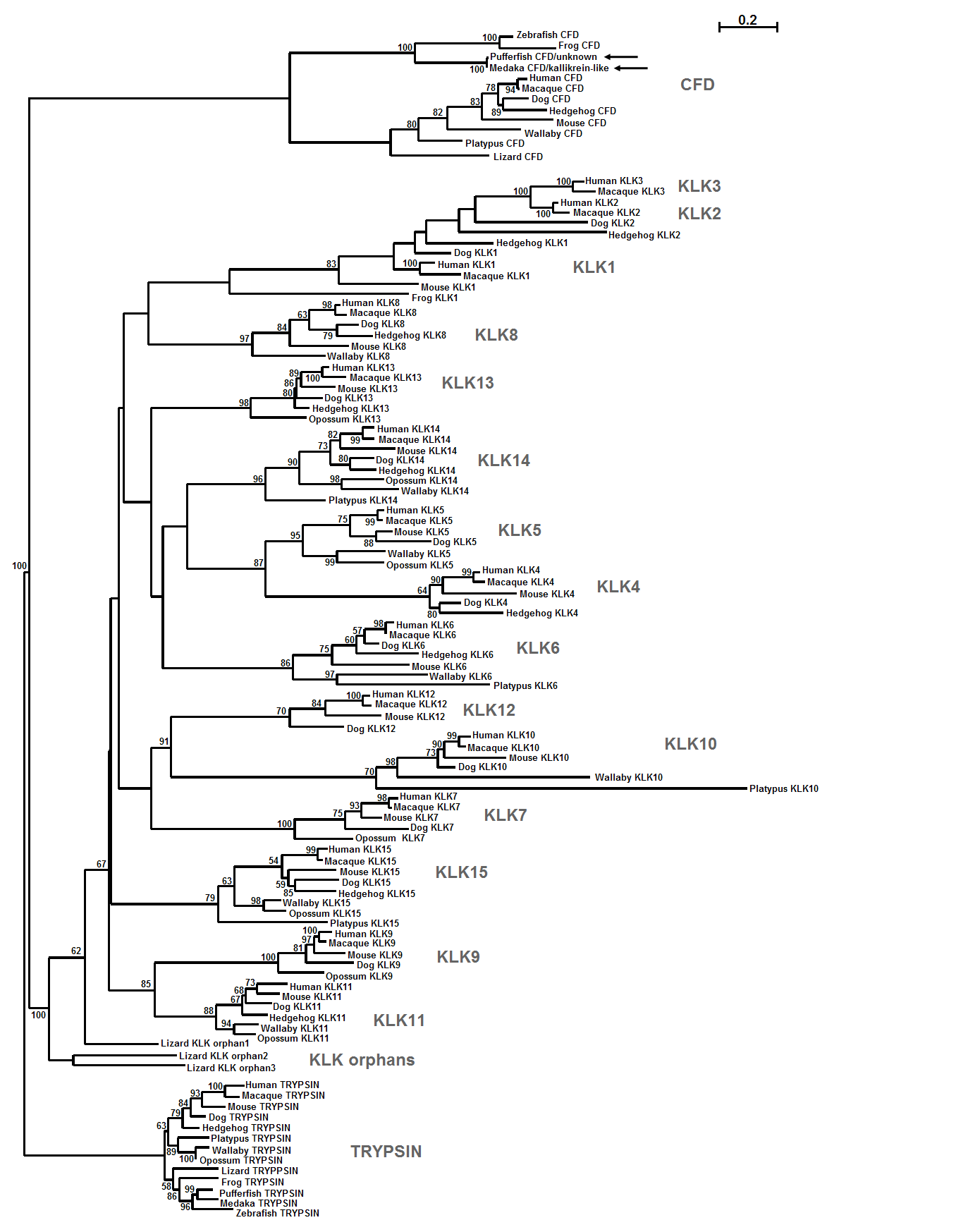

Supplement: Figure S2 — ML phylogram of KLK homologues and related proteins. The CFD/Adipsin sequences were included in the phylogenetic analysis as well. The sequences which are subject to question are indicated by arrows. Conventions are the same as in Figure 7. (0.26 MB TIF) [file pone.0013781.s002.tif]

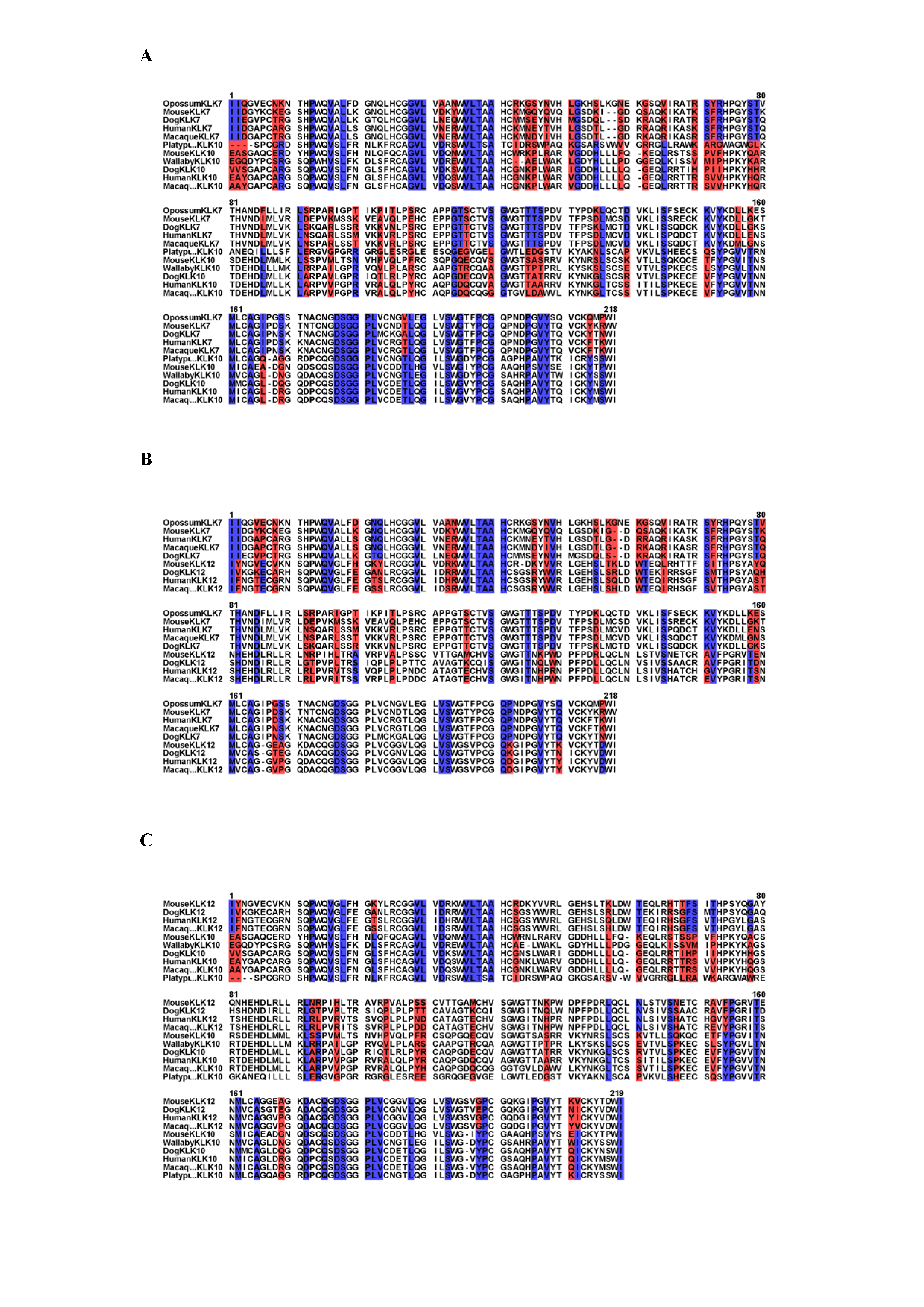

Supplement: Figure S3 — Rate shift analysis of KLK7, 10, and 12 subfamilies. The analysis further supports our phylogenetic analysis by demonstrating that KLK10 and KLK12 subfamilies are sister groups. (5.08 MB TIF) [file pone.0013781.s003.tif]
