# Supplementary material for: Evolutionary History of Tissue Kallikreins
Source: PLoS One. 2010 Nov 1;5(11):e13781. doi: 10.1371/journal.pone.0013781 (PMC2967472; doi:10.1371/journal.pone.0013781)
Supplement: Table S1 — Names and accession numbers of the sequences analyzed in the present study. The KLK pseudogene names are shown in italics. (0.32 MB DOC) [file pone.0013781.s004.doc]

| **Species** | **Sequence** | **Accession number** | **Source*** |
| --- | --- | --- | --- |
| *Homo sapiens* | Human KLK1 | P06870, NP_002248, ENSP00000301420 | 1, 2, 3 |
| *Homo sapiens* | Human KLK2 | P20151, [NP_005542](http://www.ncbi.nlm.nih.gov/entrez/query.fcgi?cmd=Retrieve&db=Protein&list_uids=5031829&dopt=GenPept&RID=97F4SSWG01P&log$=protalign&blast_rank=1), ENSP00000313581 | 1, 2, 3 |
| *Homo sapiens* | Human KLK3 | P07288, NP_001639, ENSP00000314151 | 1, 2, 3 |
| *Homo sapiens* | Human KLK4 | Q9Y5K2, NP_004908, ENSP00000326159 | 1, 2, 3 |
| *Homo sapiens* | Human KLK5 | Q9Y337, NP_036559, ENSP00000337733 | 1, 2, 3 |
| *Homo sapiens* | Human KLK6 | Q92876, NP_002765, ENSP00000309148 | 1, 2, 3 |
| *Homo sapiens* | Human KLK7 | P49862, NP_005037, ENSP00000304791 | 1, 2, 3 |
| *Homo sapiens* | Human KLK8 | O60259, NP_009127, ENSP00000291726 | 1, 2, 3 |
| *Homo sapiens* | Human KLK9 | Q9UKQ9, NP_036447, ENSP00000250366 | 1, 2, 3 |
| *Homo sapiens* | Human KLK10 | O43240, NP_002767, ENSP00000311746 | 1, 2, 3 |
| *Homo sapiens* | Human KLK11 | Q9UBX7-2, NP_006844, ENSP00000324269 | 1, 2, 3 |
| *Homo sapiens* | Human KLK12 | Q9UKR0, NP_062544, ENSP00000250351 | 1, 2, 3 |
| *Homo sapiens* | Human KLK13 | Q9UKR3, NP_056411, ENSP00000156476 | 1, 2, 3 |
| *Homo sapiens* | Human KLK14 | Q9P0G3, NP_071329, ENSP00000156499 | 1,2, 3 |
| *Homo sapiens* | Human KLK15 | Q9H2R5, NP_059979, ENSP00000301421 | 1, 2, 3 |
| *Homo sapiens* | Human Trypsin | P35030, NP_002762, ENSP00000354280 | 1, 2, 3 |
| *Homo sapiens* | Human CFD | [P00746](http://www.uniprot.org/uniprot/P00746), NP_001919, [ENSP00000332139](http://www.ensembl.org/Homo_sapiens/Transcript/ProteinSummary?db=core;g=ENSG00000197766;r=19:859665-863606;t=ENST00000327726) | 1, 2, 3 |
| *Homo sapiens* | Human Chymotrypsin | Q99895, CAA74031, ENSP00000365116 | 1, 2, 3 |
| *Gorilla gorilla* | Gorilla KLK1 | ENSGGOP00000001391 | 3 |
| *Gorilla gorilla* | Gorilla KLK3 | Q5U7M5; AAV51953; ENSGGOP00000019150 | 1, 2, 3 |
| *Gorilla gorilla* | Gorilla KLK4 | XP_524486; ENSGGOP00000006248 | 2, 3 |
| *Gorilla gorilla* | Gorilla KLK5 | AAP36503; ENSGGOP00000001008 | 2, 3 |
| *Gorilla gorilla* | Gorilla KLK6 | NP_002765; ENSGGOP00000011011 | 2, 3 |
| *Gorilla gorilla* | Gorilla KLK7 | ENSGGOP00000028139 | 3 |
| *Gorilla gorilla* | Gorilla KLK8 | Q5V9U0; ENSGGOP00000001406 | 1, 3 |
| *Gorilla gorilla* | Gorilla KLK9 | ENSGGOP00000001409 | 3 |
| *Gorilla gorilla* | Gorilla KLK10 | ENSGGOP00000001411 | 3 |
| *Gorilla gorilla* | Gorilla KLK11 | ENSGGOP00000013771 | 3 |
| *Gorilla gorilla* | Gorilla KLK12 | ENSGGOP00000028109 | 3 |
| *Gorilla gorilla* | Gorilla KLK13 | ENSGGOP00000009046 | 3 |
| *Gorilla gorilla* | Gorilla KLK14 | ENSGGOP00000001339 | 3 |
| *Gorilla gorilla* | Gorilla KLK15 | ENSGGOP00000022354 | 3 |
| *Gorilla gorilla* | Gorilla Trypsin | ENSGGOP00000012969 | 3 |
| *Homo sapiens* | Human PLG | P00747, NP_000292, ENSP00000308938 | 1, 2, 3 |
| [*Pongo pygmaeus*](http://www.ncbi.nlm.nih.gov/Taxonomy/Browser/wwwtax.cgi?mode=Info&id=9600&lvl=3&lin=f&keep=1&srchmode=1&unlock) | Orangutan KLK1 | ENSPPYP00000011528 | 3 |
| [*Pongo pygmaeus*](http://www.ncbi.nlm.nih.gov/Taxonomy/Browser/wwwtax.cgi?mode=Info&id=9600&lvl=3&lin=f&keep=1&srchmode=1&unlock) | Orangutan KLK2 | [Q3I216](http://www.uniprot.org/uniprot/Q3I216), ENSPPYP00000011533 | 1, 3 |
| [*Pongo pygmaeus*](http://www.ncbi.nlm.nih.gov/Taxonomy/Browser/wwwtax.cgi?mode=Info&id=9600&lvl=3&lin=f&keep=1&srchmode=1&unlock) | Orangutan KLK3 | [Q5U7M4](http://www.uniprot.org/uniprot/Q5U7M4), ENSPPYP00000011530 | 1, 3 |
| [*Pongo pygmaeus*](http://www.ncbi.nlm.nih.gov/Taxonomy/Browser/wwwtax.cgi?mode=Info&id=9600&lvl=3&lin=f&keep=1&srchmode=1&unlock) | Orangutan KLK5 | ENSPPYP00000011535 | 3 |
| [*Pongo pygmaeus*](http://www.ncbi.nlm.nih.gov/Taxonomy/Browser/wwwtax.cgi?mode=Info&id=9600&lvl=3&lin=f&keep=1&srchmode=1&unlock) | Orangutan KLK6 | ENSPPYP00000011536 | 3 |
| [*Pongo pygmaeus*](http://www.ncbi.nlm.nih.gov/Taxonomy/Browser/wwwtax.cgi?mode=Info&id=9600&lvl=3&lin=f&keep=1&srchmode=1&unlock) | Orangutan KLK7 | ENSPPYP00000011538 | 3 |
| [*Pongo pygmaeus*](http://www.ncbi.nlm.nih.gov/Taxonomy/Browser/wwwtax.cgi?mode=Info&id=9600&lvl=3&lin=f&keep=1&srchmode=1&unlock) | Orangutan KLK8 | ENSPPYP00000011539 | 3 |
| [*Pongo pygmaeus*](http://www.ncbi.nlm.nih.gov/Taxonomy/Browser/wwwtax.cgi?mode=Info&id=9600&lvl=3&lin=f&keep=1&srchmode=1&unlock) | Orangutan KLK9 | ENSPPYP00000011541 | 3 |
| [*Pongo pygmaeus*](http://www.ncbi.nlm.nih.gov/Taxonomy/Browser/wwwtax.cgi?mode=Info&id=9600&lvl=3&lin=f&keep=1&srchmode=1&unlock) | Orangutan KLK10 | ENSPPYP00000011542 | 3 |
| [*Pongo pygmaeus*](http://www.ncbi.nlm.nih.gov/Taxonomy/Browser/wwwtax.cgi?mode=Info&id=9600&lvl=3&lin=f&keep=1&srchmode=1&unlock) | Orangutan KLK12 | [ENSPPYP00000011543](http://www.ensembl.org/Pongo_pygmaeus/Transcript/ProteinSummary?db=core;g=ENSPPYG00000010307;r=19:52623797-52628151;t=ENSPPYT00000011986) | 3 |
| [*Pongo pygmaeus*](http://www.ncbi.nlm.nih.gov/Taxonomy/Browser/wwwtax.cgi?mode=Info&id=9600&lvl=3&lin=f&keep=1&srchmode=1&unlock) | Orangutan KLK13 | ENSPPYP00000011544 | 3 |
| [*Pongo pygmaeus*](http://www.ncbi.nlm.nih.gov/Taxonomy/Browser/wwwtax.cgi?mode=Info&id=9600&lvl=3&lin=f&keep=1&srchmode=1&unlock) | Orangutan KLK14 | ENSPPYP00000011545 | 3 |
| [*Pongo pygmaeus*](http://www.ncbi.nlm.nih.gov/Taxonomy/Browser/wwwtax.cgi?mode=Info&id=9600&lvl=3&lin=f&keep=1&srchmode=1&unlock) | Orangutan KLK15 | ENSPPYP00000011529 | 3 |
| [*Pongo pygmaeus*](http://www.ncbi.nlm.nih.gov/Taxonomy/Browser/wwwtax.cgi?mode=Info&id=9600&lvl=3&lin=f&keep=1&srchmode=1&unlock) | Orangutan Trypsin | ENSPPYP00000021436 | 3 |
| *Macaca mulatta* | Macaque KLK1 | [ENSMMUP00000008403](http://www.ensembl.org/Macaca_mulatta/Transcript/ProteinSummary?db=core;g=ENSMMUG00000006371;r=19:57061475-57077517;t=ENSMMUT00000008935) | 3 |
| *Macaca mulatta* | Macaque KLK2 | [Q3I215](http://www.uniprot.org/uniprot/Q3I215), AAZ82223, ENSMMUP00000016744 | 1, 2, 3 |
| *Macaca mulatta* | Macaque KLK3 | [P33619](http://www.uniprot.org/uniprot/P33619), CAA51957, [ENSMMUP00000031500](http://www.ensembl.org/Macaca_mulatta/Transcript/ProteinSummary?db=core;g=ENSMMUG00000012841;r=19:57061475-57067329;t=ENSMMUT00000038399) | 1, 2, 3 |
| *Macaca mulatta* | Macaque KLK4 | XP_001116184, [ENSMMUP00000031498](http://www.ensembl.org/Macaca_mulatta/Transcript/ProteinSummary?db=core;g=ENSMMUG00000028729;r=19:57061475-57077517;t=ENSMMUT00000038396) | 2, 3 |
| *Macaca mulatta* | Macaque KLK5 | XP_001116247, ENSMMUP00000031497 | 2, 3 |
| *Macaca mulatta* | Macaque KLK6 | XP_001114451, ENSMMUP00000031496 | 2, 3 |
| *Macaca mulatta* | Macaque KLK7 | XP_001118798, ENSMMUP00000041345 | 2, 3 |
| *Macaca mulatta* | Macaque KLK8 | A6YBE7, ABQ81883, ENSMMUP00000020253 | 1, 2, 3 |
| *Macaca mulatta* | Macaque KLK9 | ENSMMUP00000020260 | 3 |
| *Macaca mulatta* | Macaque KLK10 | XP_001114486, ENSMMUP00000020263 | 2, 3 |
| *Macaca mulatta* | Macaque KLK12 | XP_001116273, ENSMMUP00000031493 | 2, 3 |
| *Macaca mulatta* | Macaque KLK13 | ENSMMUP00000031491 | 3 |
| *Macaca mulatta* | Macaque KLK14 | ENSMMUP00000036982 | 3 |
| *Macaca mulatta* | Macaque KLK15 | XP_001116199, [ENSMMUP00000002661](http://www.ensembl.org/Macaca_mulatta/Transcript/ProteinSummary?db=core;g=ENSMMUG00000001991;r=19:57021470-57022813;t=ENSMMUT00000002817) | 2, 3 |
| *Macaca mulatta* | Macaque Trypsin | **Q5H728;** AC149201, [ENSMMUP00000000368](http://www.ensembl.org/Macaca_mulatta/Transcript/ProteinSummary?db=core;g=ENSMMUG00000001991;r=19:57021470-57022813;t=ENSMMUT00000002817) | 1, 2, 3 |
| *Macaca mulatta* | Macaque CFD | XP_001117186, ENSMMUP00000012662 | 2, 3 |
| *Callithrix jacchus* | Marmoset KLK1 | ENSCJAP00000030675 | 3 |
| *Callithrix jacchus* | Marmoset KLK2 | ENSCJAP00000030661 | 3 |
| *Callithrix jacchus* | Marmoset KLK4 | ENSCJAP00000030628 | 3 |
| *Callithrix jacchus* | Marmoset KLK6 | ENSCJAP00000030592 | 3 |
| *Callithrix jacchus* | Marmoset KLK7 | ENSCJAP00000030573 | 3 |
| *Callithrix jacchus* | Marmoset KLK8 | ENSCJAP00000011525 | 3 |
| *Callithrix jacchus* | Marmoset KLK9 | ENSCJAP00000011569 | 3 |
| *Callithrix jacchus* | Marmoset KLK10 | ENSCJAP00000011593 | 3 |
| *Callithrix jacchus* | Marmoset KLK11 | ENSCJAP00000011645 | 3 |
| *Callithrix jacchus* | Marmoset KLK12 | ENSCJAP00000011679 | 3 |
| *Callithrix jacchus* | Marmoset KLK13 | ENSCJAP00000011520 | 3 |
| *Callithrix jacchus* | Marmoset KLK14 | ENSCJAP00000038588 | 3 |
| *Callithrix jacchus* | Marmoset KLK15 | ENSCJAP00000030677 | 3 |
| *Callithrix jacchus* | Marmoset Trypsin | ENSCJAP00000025430 | 3 |
| *Microcebus murinus* | Mouse Lemur KLK2 | ENSMICP00000001577 | 3 |
| *Microcebus murinus* | Mouse Lemur KLK4 | ENSMICP00000010768 | 3 |
| *Microcebus murinus* | Mouse Lemur KLK5 | ENSMICP00000015382 | 3 |
| *Microcebus murinus* | Mouse Lemur KLK8 | ENSMICP00000015385 | 3 |
| *Microcebus murinus* | Mouse Lemur KLK10 | ENSMICP00000015390 | 3 |
| *Microcebus murinus* | Mouse Lemur KLK11 | ENSMICP00000015394 | 3 |
| *Microcebus murinus* | Mouse Lemur KLK12 | ENSMICP00000015399 | 3 |
| *Microcebus murinus* | Mouse Lemur KLK13 | ENSMICP00000015404 | 3 |
| *Microcebus murinus* | Mouse Lemur KLK15 | ENSMICP00000000475 | 3 |
| *Microcebus murinus* | Mouse Lemur Trypsin | ENSMICP00000006691 | 3 |
| *Bos taurus* | Cattle KLK1 | [Q6H320](http://www.uniprot.org/uniprot/Q6H320), NP_001008416, ENSBTAP00000024677 | 1, 2, 3 |
| *Bos taurus* | Cattle KLK4 | XP_584478, ENSBTAP00000027207 | 2, 3 |
| *Bos taurus* | Cattle KLK5 | XP_875061, ENSBTAP00000027772 | 2, 3 |
| *Bos taurus* | Cattle KLK6 | [A6QQ95](http://www.uniprot.org/uniprot/A6QQ95), NP_001094610, ENSBTAP00000020121 | 1, 2, 3 |
| *Bos taurus* | Cattle KLK7 | [Q08D90](http://www.uniprot.org/uniprot/Q08D90), NP_001068724, ENSBTAP00000020123 | 1, 2, 3 |
| *Bos taurus* | Cattle KLK8 | XP_875861, ENSBTAP00000020127 | 2, 3 |
| *Bos taurus* | Cattle KLK9 | XP_609272, ENSBTAP00000050470 | 2, 3 |
| *Bos taurus* | Cattle KLK10 | [Q0VCZ4](http://www.uniprot.org/uniprot/Q0VCZ4), NP_001069358, ENSBTAP00000031726 | 1, 2, 3 |
| *Bos taurus* | Cattle KLK11 | XP_605111, ENSBTAP00000002912 | 2, 3 |
| *Bos taurus* | Cattle KLK12 | XP_875874, ENSBTAP00000002923 | 2, 3 |
| *Bos taurus* | Cattle KLK13 | XP_593029, ENSBTAP00000012346 | 2, 3 |
| *Bos taurus* | Cattle KLK14 | XP_870367, ENSBTAP00000016644 | 2, 3 |
| *Bos taurus* | Cattle KLK15 | XP_001789828, ENSBTAP00000024680 | 2, 3 |
| *Bos taurus* | Cattle Trypsin | Q547S4, NP_777115, ENSBTAP00000028731 | 1, 2, 3 |
| *Tursiops truncatus* | Dolphin KLK1 | ENSTTRP00000010502 | 3 |
| *Tursiops truncatus* | Dolphin KLK4 | ENSTTRP00000005417 | 3 |
| *Tursiops truncatus* | Dolphin KLK5 | ENSTTRP00000005415 | 3 |
| *Tursiops truncatus* | Dolphin KLK6 | ENSTTRP00000005414 | 3 |
| *Tursiops truncatus* | Dolphin KLK8 | ENSTTRP00000005413 | 3 |
| *Tursiops truncatus* | Dolphin KLK9 | ENSTTRP00000005412 | 3 |
| *Tursiops truncatus* | Dolphin KLK10 | ENSTTRP00000000847 | 3 |
| *Tursiops truncatus* | Dolphin KLK11 | ENSTTRP00000000846 | 3 |
| *Tursiops truncatus* | Dolphin KLK12 | ENSTTRP00000000841 | 3 |
| *Tursiops truncatus* | Dolphin KLK14 | ENSTTRP00000000840 | 3 |
| *Tursiops truncatus* | Dolphin Trypsin | ENSTTRP00000012797 | 3 |
| *Equus caballus* | Horse KLK1 | **Q6H322,** NP_001075361, ENSECAP00000018835 | 1, 2, 3 |
| *Equus caballus* | Horse KLK2 | **Q6H321,** NP_001075362, **ENSECAP00000009529** | 1, 2, 3 |
| *Equus caballus* | Horse KLK4 | XP_001497570, ENSECAP00000016324 | 2, 3 |
| *Equus caballus* | Horse KLK5 | XP_001917941, ENSECAP00000021961 | 2, 3 |
| *Equus caballus* | Horse KLK6 | XP_001917944, ENSECAP00000022280 | 2, 3 |
| *Equus caballus* | Horse KLK8 | XP_001497439, ENSECAP00000001044 | 2, 3 |
| *Equus caballus* | Horse KLK9 | XP_001497351, ENSECAP00000006463 | 2, 3 |
| *Equus caballus* | Horse KLK11 | XP_001917514, ENSECAP00000007261 | 2, 3 |
| *Equus caballus* | Horse KLK12 | XP_001497277, ENSECAP00000011285 | 2, 3 |
| *Equus caballus* | Horse KLK13 | XP_001497240, ENSECAP00000011741 | 2, 3 |
| *Equus caballus* | Horse KLK14 | XP_001497219, ENSECAP00000013145 | 2, 3 |
| *Equus caballus* | Horse KLK15 | XP_001917492, ENSECAP00000007410 | 2, 3 |
| *Equus caballus* | Horse Trypsin | XP_001489596, ENSECAP00000013655 | 2, 3 |
| *Pteropus vampyrus* | Megabat KLK1 | ENSPVAP00000003241 | 3 |
| *Pteropus vampyrus* | Megabat KLK5 | ENSPVAP00000009805 | 3 |
| *Pteropus vampyrus* | Megabat KLK6 | ENSPVAP00000009806 | 3 |
| *Pteropus vampyrus* | Megabat KLK7 | ENSPVAP00000009807 | 3 |
| *Pteropus vampyrus* | Megabat KLK8 | ENSPVAP00000009808 | 3 |
| *Pteropus vampyrus* | Megabat KLK9 | ENSPVAP00000009809 | 3 |
| *Pteropus vampyrus* | Megabat KLK10 | ENSPVAP00000009810 | 3 |
| *Pteropus vampyrus* | Megabat KLK11 | ENSPVAP00000009811 | 3 |
| *Pteropus vampyrus* | Megabat KLK12 | ENSPVAP00000009812 | 3 |
| *Pteropus vampyrus* | Megabat KLK13 | ENSPVAP00000009813 | 3 |
| *Pteropus vampyrus* | Megabat KLK14 | ENSPVAP00000009814 | 3 |
| *Pteropus vampyrus* | Megabat KLK15 | ENSPVAP00000003237 | 3 |
| *Pteropus vampyrus* | Megabat Trypsin | [ENSPVAP00000004342](http://www.ensembl.org/Pteropus_vampyrus/Transcript/ProteinSummary?db=core;g=ENSPVAG00000004590;r=scaffold_7685:8031-11385;t=ENSPVAT00000004588) | 3 |
| *Canis familiaris* | Dog KLK1 | **Q29474,** CAA53210, ENSCAFP00000004315 | 1, 2, 3 |
| *Canis familiaris* | Dog KLK2 | XP_854598, ENSCAFP00000033340 | 2, 3 |
| *Canis familiaris* | Dog KLK4 | XP_541470, ENSCAFP00000004303 | 2, 3 |
| *Canis familiaris* | Dog KLK5 | XP_541469, ENSCAFP00000004293 | 2, 3 |
| *Canis familiaris* | Dog KLK6 | XP_533605, ENSCAFP00000033419 | 2, 3 |
| *Canis familiaris* | Dog KLK7 | XP_854584, ENSCAFP00000004287 | 2, 3 |
| *Canis familiaris* | Dog KLK8 | XP_541468, ENSCAFP00000004284 | 2, 3 |
| *Canis familiaris* | Dog KLK9 | ENSCAFP00000004278 | 3 |
| *Canis familiaris* | Dog KLK10 | XP_541467, ENSCAFP00000004273 | 2, 3 |
| *Canis familiaris* | Dog KLK11 | XP_533604, ENSCAFP00000004266 | 2, 3 |
| *Canis familiaris* | Dog KLK12 | XP_854572, ENSCAFP00000004265 | 2, 3 |
| *Canis familiaris* | Dog KLK13 | XP_541465, ENSCAFP00000004264 | 2, 3 |
| *Canis familiaris* | Dog KLK14 | **C7T1J3,** ACU82847, ENSCAFP00000033624 | 1, 2, 3 |
| *Canis familiaris* | Dog KLK15 | XP_537938, ENSCAFP00000004313 | 2, 3 |
| *Canis familiaris* | Dog Trypsin | **P06872,** XP_532744, **ENSCAFP00000021363** | 1, 2, 3 |
| *Canis familiaris* | Dog CFD | **XP_542213, ENSMEUP00000010890** | 2, 3 |
| *Felis catus* | Cat KLK1 | ENSFCAP00000007724 | 3 |
| *Felis catus* | Cat KLK2 | ENSFCAP00000007656 | 3 |
| *Felis catus* | Cat KLK4 | ENSFCAP00000007658 | 3 |
| *Felis catus* | Cat KLK5 | ENSFCAP00000007660 | 3 |
| *Felis catus* | Cat KLK6 | ENSFCAP00000007662 | 3 |
| *Felis catus* | Cat KLK9 | ENSFCAP00000015358 | 3 |
| *Felis catus* | Cat KLK11 | ENSFCAP00000007664 | 3 |
| *Felis catus* | Cat KLK12 | ENSFCAP00000007666 | 3 |
| *Felis catus* | Cat KLK13 | ENSFCAP00000000144 | 3 |
| *Felis catus* | Cat KLK15 | ENSFCAP00000006034 | 3 |
| *Felis catus* | Cat Trypsin | ENSFCAP00000011020 | 3 |
| *Erinaceus europaeus* | [Hedgehog](http://www.ensembl.org/Erinaceus_europaeus/Info/Index/) KLK1 | ENSEEUP00000002782 | 3 |
| *Erinaceus europaeus* | [Hedgehog](http://www.ensembl.org/Erinaceus_europaeus/Info/Index/) KLK2 | ENSEEUP00000011887 | 3 |
| *Erinaceus europaeus* | [Hedgehog](http://www.ensembl.org/Erinaceus_europaeus/Info/Index/) KLK4 | ENSEEUP00000011954 | 3 |
| *Erinaceus europaeus* | [Hedgehog](http://www.ensembl.org/Erinaceus_europaeus/Info/Index/) KLK6 | ENSEEUP00000010483 | 3 |
| *Erinaceus europaeus* | [Hedgehog](http://www.ensembl.org/Erinaceus_europaeus/Info/Index/) KLK8 | ENSEEUP00000010388 | 3 |
| *Erinaceus europaeus* | [Hedgehog](http://www.ensembl.org/Erinaceus_europaeus/Info/Index/) KLK11 | ENSEEUP00000009367 | 3 |
| *Erinaceus europaeus* | [Hedgehog](http://www.ensembl.org/Erinaceus_europaeus/Info/Index/) KLK13 | ENSEEUP00000007538 | 3 |
| *Erinaceus europaeus* | [Hedgehog](http://www.ensembl.org/Erinaceus_europaeus/Info/Index/) KLK14 | ENSEEUP00000000275 | 3 |
| *Erinaceus europaeus* | [Hedgehog](http://www.ensembl.org/Erinaceus_europaeus/Info/Index/) KLK15 | ENSEEUP00000002725 | 3 |
| *Erinaceus europaeus* | [Hedgehog](http://www.ensembl.org/Erinaceus_europaeus/Info/Index/) Trypsin | ENSEEUP00000004013 | 3 |
| *Erinaceus europaeus* | Hedgehog CFD | ENSEEUP00000011879 | 3 |
| *Procavia capensis* | Hyrax KLK1 | ENSPCAP00000008956 | 3 |
| *Procavia capensis* | Hyrax KLK5 | ENSPCAP00000004303 | 3 |
| *Procavia capensis* | Hyrax KLK7 | ENSPCAP00000001154 | 3 |
| *Procavia capensis* | Hyrax KLK8 | ENSPCAP00000001200 | 3 |
| *Procavia capensis* | Hyrax KLK9 | ENSPCAP00000001223 | 3 |
| *Procavia capensis* | Hyrax KLK10 | ENSPCAP00000001276 | 3 |
| *Procavia capensis* | Hyrax KLK11 | ENSPCAP00000001322 | 3 |
| *Procavia capensis* | Hyrax KLK12 | ENSPCAP00000001345 | 3 |
| *Procavia capensis* | Hyrax KLK14 | ENSPCAP00000005504 | 3 |
| *Procavia capensis* | Hyrax KLK15 | ENSPCAP00000008975 | 3 |
| *Procavia capensis* | Hyrax Trypsin | ENSPCAP00000009040 | 3 |
| *Echinops telfairi* | Tenrec KLK1 | ENSETEP00000009366 | 3 |
| *Echinops telfairi* | Tenrec KLK8 | ENSETEP00000012638 | 3 |
| *Echinops telfairi* | Tenrec KLK10 | ENSETEP00000008094 | 3 |
| *Echinops telfairi* | Tenrec KLK11 | ENSETEP00000012639 | 3 |
| *Echinops telfairi* | Tenrec KLK15 | ENSETEP00000004184 | 3 |
| *Echinops telfairi* | Tenrec Trypsin | ENSETEP00000007412 | 3 |
| *Mus musculus* | Mouse Klk1 | P15947, NP_034769, ENSMUSP00000074659 | 1, 2, 3 |
| *Mus musculus* | *Mouse Klk2-ps* | AY152430.1 | 2 |
| *Mus musculus* | Mouse Klk1b1 | P00755, NP_034775, ENSMUSP00000077879 | 1, 2, 3 |
| *Mus musculus* | Mouse Klk1b3 | P00756, NP_032719, ENSMUSP00000082577 | 1, 2, 3 |
| *Mus musculus* | Mouse Klk1b4 | P00757, NP_035045, ENSMUSP00000076576 | 1, 2, 3 |
| *Mus musculus* | Mouse Klk1b5 | P15945, NP_032482, ENSMUSP00000073964 | 1, 2, 3 |
| *Mus musculus* | Mouse Klk1b8 | P07628, NP_032483, ENSMUSP00000072063 | 1, 2, 3 |
| *Mus musculus* | Mouse Klk1b9 | P15949, NP_034246, ENSMUSP00000080133 | 1, 2, 3 |
| *Mus musculus* | Mouse Klk1b11 | P15946, NP_034770, ENSMUSP00000007156 | 1, 2, 3 |
| *Mus musculus* | Mouse Klk1b16 | P04071, NP_032480, ENSMUSP00000005933 | 1, 2, 3 |
| *Mus musculus* | Mouse Klk1b21 | Q61759, NP_034772, ENSMUSP00000082582 | 1, 2, 3 |
| *Mus musculus* | Mouse Klk1b22 | P15948, NP_034244, ENSMUSP00000076733 | 1, 2, 3 |
| *Mus musculus* | Mouse Klk1b24 | Q61754, NP_034773, ENSMUSP00000073392 | 1, 2, 3 |
| *Mus musculus* | Mouse Klk1b26 | P36369, NP_034774, ENSMUSP00000047488 | 1, 2, 3 |
| *Mus musculus* | Mouse Klk1b27 | Q9JM71, NP_064664, ENSMUSP00000078786 | 1, 2, 3 |
| *Mus musculus* | Mouse Klk4 | Q9JIS2, NP_064312, ENSMUSP00000007161 | 1, 2, 3 |
| *Mus musculus* | Mouse Klk5 | [Q9D140](http://www.uniprot.org/uniprot/Q9D140) , NP_081082, ENSMUSP00000049339 | 1, 2, 3 |
| *Mus musculus* | Mouse Klk6 | [Q91Y82](http://www.uniprot.org/uniprot/Q91Y82), NP_035307, ENSMUSP00000103600 | 1, 2, 3 |
| *Mus musculus* | Mouse Klk7 | Q91VE3, NP_036002, ENSMUSP00000032955 | 1, 2, 3 |
| *Mus musculus* | Mouse Klk8 | [Q61955](http://www.uniprot.org/uniprot/Q61955), NP_032966, ENSMUSP00000082588 | 1, 2, 3 |
| *Mus musculus* | Mouse Klk9 | [Q32M27](http://www.uniprot.org/uniprot/Q32M27), NP_082936, ENSMUSP00000005891 | 1, 2, 3 |
| *Mus musculus* | Mouse Klk10 | [Q99M20](http://www.uniprot.org/uniprot/Q99M20), NP_598473, ENSMUSP00000014058 | 1, 2, 3 |
| *Mus musculus* | Mouse Klk11 | [Q9QYN3](http://www.uniprot.org/uniprot/Q9QYN3), NP_064358, ENSMUSP00000079101 | 1, 2, 3 |
| *Mus musculus* | Mouse Klk12 | [B2RVZ0](http://www.uniprot.org/uniprot/B2RVZ0), NP_081373, ENSMUSP00000103604 | 1, 2, 3 |
| *Mus musculus* | Mouse Klk13 | [Q8CGR6](http://www.uniprot.org/uniprot/Q8CGR6), NP_001034131, ENSMUSP00000065308 | 1, 2, 3 |
| *Mus musculus* | Mouse Klk14 | [Q8CGR5](http://www.uniprot.org/uniprot/Q8CGR5), NP_777355, ENSMUSP00000056935 | 1, 2, 3 |
| *Mus musculus* | Mouse Klk15 | [Q8CGR4](http://www.uniprot.org/uniprot/Q8CGR4), NP_777354, ENSMUSP00000066969 | 1, 2, 3 |
| *Mus musculus* | Mouse Trypsin | Q792Y9, NP_001034086, ENSMUSP00000039684 | 1, 2, 3 |
| *Mus musculus* | Mouse CFD | P03953, NP_038487, ENSMUSP00000056836 | 1, 2, 3 |
| *Rattus norvegicus* | Rat Klk1 | **P00758,** [AAA41464](http://www.ebi.ac.uk/cgi-bin/dbfetch?db=emblcds&id=AAA41464), ENSRNOP00000025831 | 1, 2, 3 |
| *Rattus norvegicus* | *Rat Klk2-ps* | **BK001374** | 2 |
| *Rattus norvegicus* | Rat Klk1c2 | **P00759,** AAA41466, ENSRNOP00000025701 | 1, 2, 3 |
| *Rattus norvegicus* | Rat Klk1c3 | **P15950,** AAA41465, ENSRNOP00000025777 | 1, 2, 3 |
| *Rattus norvegicus* | Rat Klk1c4 | **Q63274,** AAA58781, ENSRNOP00000050040 | 1, 2, 3 |
| *Rattus norvegicus* | Rat Klk1c6 | Q6IE61, CAE48387, ENSRNOP00000049589 | 1, 2, 3 |
| *Rattus norvegicus* | Rat Klk1c7 | **P36373,** AAA41461, ENSRNOP00000025750 | 1, 2, 3 |
| *Rattus norvegicus* | Rat Klk1c8 | **P36374,** AAA42036, ENSRNOP00000025715 | 1, 2, 3 |
| *Rattus norvegicus* | Rat Klk1c9 | **P07647,** AAA41467, ENSRNOP00000025723 | 1, 2, 3 |
| *Rattus norvegicus* | Rat Klk1c10 | **P36375,** AAB24071, ENSRNOP00000048581 | 1, 2, 3 |
| *Rattus norvegicus* | Rat Klk1c12 | **P36376,** AAA51640, ENSRNOP00000025631 | 1, 2, 3 |
| *Rattus norvegicus* | Rat Klk4 | **Q6IE12,** CAE51907, ENSRNOP00000025614 | 1, 2, 3 |
| *Rattus norvegicus* | Rat Klk5 | XP_001074943, ENSRNOP00000025489 | 2, 3 |
| *Rattus norvegicus* | Rat Klk6 | **O54854,** AAC02300, ENSRNOP00000060676 | 1, 2, 3 |
| *Rattus norvegicus* | Rat Klk7 | NP_001099724, ENSRNOP00000025299 | 2, 3 |
| *Rattus norvegicus* | Rat Klk8 | **O88780,** CAA06643, ENSRNOP00000025174 | 1, 2, 3 |
| *Rattus norvegicus* | Rat Klk9 | NP_001099723, ENSRNOP00000034824 | 2, 3 |
| *Rattus norvegicus* | Rat Klk10 | **Q6IE55,** [CAE48393](http://www.ebi.ac.uk/cgi-bin/dbfetch?db=emblcds&id=CAE48393), ENSRNOP00000043107 | 1, 2, 3 |
| *Rattus norvegicus* | Rat Klk11 | NP_001099722, ENSRNOP00000025055 | 2, 3 |
| *Rattus norvegicus* | Rat Klk12 | NP_001100978, ENSRNOP00000024758 | 2, 3 |
| *Rattus norvegicus* | Rat Klk13 | XP_218643, ENSRNOP00000032820 | 2, 3 |
| *Rattus norvegicus* | Rat Klk14 | XP_218641, ENSRNOP00000032634 | 2, 3 |
| *Rattus norvegicus* | Rat Klk15 | XP_001080538, ENSRNOP00000037756 | 2, 3 |
| *Rattus norvegicus* | Rat Trypsin | **P00762,** AAA98518, ENSRNOP00000018853 | 1, 2, 3 |
| [*Dipodomys ordii*](http://www.ensembl.org/Homo_sapiens/Search/Details?_C=eJyLz2FIzWOIL8tjSElNSyzNKWGIL2Rw9Qt28Q8KMIAAC0szI4Xk*KKC*KLEklT9kqLUVCulkPwC*eD80qLkVP1UUzMlhviMzBK30pwcBkMDBgBCVRmR&_c=%2B12328842104476958995) | Kangaroo Rat KLK1 | ENSDORP00000008962 | 3 |
| [*Dipodomys ordii*](http://www.ensembl.org/Homo_sapiens/Search/Details?_C=eJyLz2FIzWOIL8tjSElNSyzNKWGIL2Rw9Qt28Q8KMIAAC0szI4Xk*KKC*KLEklT9kqLUVCulkPwC*eD80qLkVP1UUzMlhviMzBK30pwcBkMDBgBCVRmR&_c=%2B12328842104476958995) | Kangaroo Rat KLK2 | ENSDORP00000011773 | 3 |
| [*Dipodomys ordii*](http://www.ensembl.org/Homo_sapiens/Search/Details?_C=eJyLz2FIzWOIL8tjSElNSyzNKWGIL2Rw9Qt28Q8KMIAAC0szI4Xk*KKC*KLEklT9kqLUVCulkPwC*eD80qLkVP1UUzMlhviMzBK30pwcBkMDBgBCVRmR&_c=%2B12328842104476958995) | Kangaroo Rat KLK5 | ENSDORP00000005927 | 3 |
| [*Dipodomys ordii*](http://www.ensembl.org/Homo_sapiens/Search/Details?_C=eJyLz2FIzWOIL8tjSElNSyzNKWGIL2Rw9Qt28Q8KMIAAC0szI4Xk*KKC*KLEklT9kqLUVCulkPwC*eD80qLkVP1UUzMlhviMzBK30pwcBkMDBgBCVRmR&_c=%2B12328842104476958995) | Kangaroo Rat KLK7 | ENSDORP00000001675 | 3 |
| [*Dipodomys ordii*](http://www.ensembl.org/Homo_sapiens/Search/Details?_C=eJyLz2FIzWOIL8tjSElNSyzNKWGIL2Rw9Qt28Q8KMIAAC0szI4Xk*KKC*KLEklT9kqLUVCulkPwC*eD80qLkVP1UUzMlhviMzBK30pwcBkMDBgBCVRmR&_c=%2B12328842104476958995) | Kangaroo Rat KLK8 | ENSDORP00000001674 | 3 |
| [*Dipodomys ordii*](http://www.ensembl.org/Homo_sapiens/Search/Details?_C=eJyLz2FIzWOIL8tjSElNSyzNKWGIL2Rw9Qt28Q8KMIAAC0szI4Xk*KKC*KLEklT9kqLUVCulkPwC*eD80qLkVP1UUzMlhviMzBK30pwcBkMDBgBCVRmR&_c=%2B12328842104476958995) | Kangaroo Rat KLK9 | ENSDORP00000001671 | 3 |
| [*Dipodomys ordii*](http://www.ensembl.org/Homo_sapiens/Search/Details?_C=eJyLz2FIzWOIL8tjSElNSyzNKWGIL2Rw9Qt28Q8KMIAAC0szI4Xk*KKC*KLEklT9kqLUVCulkPwC*eD80qLkVP1UUzMlhviMzBK30pwcBkMDBgBCVRmR&_c=%2B12328842104476958995) | Kangaroo Rat KLK11 | ENSDORP00000001664 | 3 |
| [*Dipodomys ordii*](http://www.ensembl.org/Homo_sapiens/Search/Details?_C=eJyLz2FIzWOIL8tjSElNSyzNKWGIL2Rw9Qt28Q8KMIAAC0szI4Xk*KKC*KLEklT9kqLUVCulkPwC*eD80qLkVP1UUzMlhviMzBK30pwcBkMDBgBCVRmR&_c=%2B12328842104476958995) | Kangaroo Rat KLK13 | ENSDORP00000000651 | 3 |
| [*Dipodomys ordii*](http://www.ensembl.org/Homo_sapiens/Search/Details?_C=eJyLz2FIzWOIL8tjSElNSyzNKWGIL2Rw9Qt28Q8KMIAAC0szI4Xk*KKC*KLEklT9kqLUVCulkPwC*eD80qLkVP1UUzMlhviMzBK30pwcBkMDBgBCVRmR&_c=%2B12328842104476958995) | Kangaroo Rat KLK15 | ENSDORP00000008961 | 3 |
| [*Dipodomys ordii*](http://www.ensembl.org/Homo_sapiens/Search/Details?_C=eJyLz2FIzWOIL8tjSElNSyzNKWGIL2Rw9Qt28Q8KMIAAC0szI4Xk*KKC*KLEklT9kqLUVCulkPwC*eD80qLkVP1UUzMlhviMzBK30pwcBkMDBgBCVRmR&_c=%2B12328842104476958995) | Kangaroo Rat Trypsin | ENSDORP00000008157 | 3 |
| *Dasypus novemcinctus* | Armadillo KLK5 | ENSDNOP00000010100 | 3 |
| *Dasypus novemcinctus* | Armadillo KLK8 | ENSDNOP00000010106 | 3 |
| *Dasypus novemcinctus* | Armadillo KLK9 | ENSDNOP00000015844 | 3 |
| *Dasypus novemcinctus* | Armadillo KLK10 | ENSDNOP00000008053 | 3 |
| *Dasypus novemcinctus* | Armadillo KLK15 | ENSDNOP00000016489 | 3 |
| *Dasypus novemcinctus* | Armadillo Trypsin | ENSDNOP00000016956 | 3 |
| *Monodelphis domestica* | Opossum KLK5 | XP_001366980, ENSMODP00000008050 | 2, 3 |
| *Monodelphis domestica* | Opossum KLK7 | XP_001366782, ENSMODP00000008063 | 2, 3 |
| *Monodelphis domestica* | Opossum KLK9 | XP_001366407, ENSMODP00000008073 | 2, 3 |
| *Monodelphis domestica* | Opossum KLK11 | XP_001366348, ENSMODP00000008111 | 2, 3 |
| *Monodelphis domestica* | Opossum KLK13 | XP_001378281, ENSMODP00000026032 | 2, 3 |
| *Monodelphis domestica* | Opossum KLK14 | XP_001378295, ENSMODP00000026033 | 2, 3 |
| *Monodelphis domestica* | Opossum KLK15 | XP_001367028, ENSMODP00000008019 | 2, 3 |
| *Monodelphis domestica* | Opossum Trypsin | XP_001362226, ENSMODP00000000799 | 2, 3 |
| *Macropus eugenii* | Wallaby KLK5 | ENSMEUP00000000346 | 3 |
| *Macropus eugenii* | Wallaby KLK6 | ENSMEUP00000000353 | 3 |
| *Macropus eugenii* | Wallaby KLK8 | ENSMEUP00000001148 | 3 |
| *Macropus eugenii* | Wallaby KLK10 | ENSMEUP00000012875 | 3 |
| *Macropus eugenii* | Wallaby KLK11 | ENSMEUP00000012867 | 3 |
| *Macropus eugenii* | Wallaby KLK14 | ENSMEUP00000001798 | 3 |
| *Macropus eugenii* | Wallaby KLK15 | ENSMEUP00000002910 | 3 |
| *Macropus eugenii* | Wallaby Trypsin | ENSMEUP00000010977 | 3 |
| *Macropus eugenii* | Wallaby CFD | ENSMEUP00000010890 | 3 |
| *Ornithorhynchus anatinus* | Platypus KLK5L | XP_001521228 | 2 |
| *Ornithorhynchus anatinus* | Platypus KLK6 | XP_001519630, ENSOANP00000017799 | 2, 3 |
| *Ornithorhynchus anatinus* | Platypus KLK10 | XP_001516342, ENSOANP00000021629 | 2, 3 |
| *Ornithorhynchus anatinus* | Platypus KLK14 | XP_001521157, ENSOANP00000010185 | 2, 3 |
| *Ornithorhynchus anatinus* | Platypus KLK15 | XP_001521284, ENSOANP00000017133 | 2, 3 |
| *Ornithorhynchus anatinus* | Platypus Trypsin | XP_001518130, [ENSOANP00000014158](http://www.ensembl.org/Ornithorhynchus_anatinus/Transcript/ProteinSummary?db=core;g=ENSOANG00000008891;r=Contig9404:15014-24357;t=ENSOANT00000014161) | 2, 3 |
| *Ornithorhynchus anatinus* | Platypus CFD | **Q9GMD8**, AAG00454, [ENSOANP00000023866](http://www.ensembl.org/Ornithorhynchus_anatinus/Transcript/ProteinSummary?db=core;g=ENSOANG00000015156;r=Contig25868:5610-10689;t=ENSOANT00000023870) | 1, 2, 3 |
| *Taeniopygia guttata* | Zebrafinch KLK-like^t^ | XP_002190734, ENSTGUP00000014818 | 2, 3 |
| *Meleagris gallopavo* | Turkey KLK-like^t^ | **ENSMGAP00000005910** | 3 |
| *Anolis carolinensis* | Lizard KLK orphan1 | ENSACAP00000016470 | 3 |
| *Anolis carolinensis* | Lizard KLK orphan2 | ENSACAP00000016474 | 3 |
| *Anolis carolinensis* | Lizard KLK orphan3 | ENSACAP00000016476 | 3 |
| *Anolis carolinensis* | Lizard KLKL toxin | ENSACAP00000005461 | 3 |
| *Anolis carolinensis* | Lizard TRYPSIN | [ENSACAP00000007752](http://www.ensembl.org/Anolis_carolinensis/Transcript/ProteinSummary?db=core;g=ENSACAG00000007893;r=scaffold_128:40822-46539;t=ENSACAT00000007918) | 3 |
| *Anolis carolinensis* | Lizard CFD | [ENSACAP00000005210](http://www.ensembl.org/Anolis_carolinensis/Transcript/ProteinSummary?db=core;g=ENSACAG00000005334;r=scaffold_1647:38505-39000;t=ENSACAT00000005327) | 3 |
| *Xenopus tropicalis* | Frog KLK1 | Q5M908, AAH87753, ENSXETP00000006440 | 1, 2, 3 |
| *Xenopus tropicalis* | Frog TRYPSIN | [Q5M959](http://www.uniprot.org/uniprot/Q5M959), NP_001011199, [ENSXETP00000016876](http://www.ensembl.org/Xenopus_tropicalis/Transcript/ProteinSummary?db=core;g=ENSXETG00000007733;r=scaffold_1481:27913-29699;t=ENSXETT00000016876) | 1, 2, 3 |
| *Xenopus tropicalis* | Frog CFD | [A9UMF4](http://www.uniprot.org/uniprot/A9UMF4), NP_001107486, [ENSXETP00000046776](http://www.ensembl.org/Xenopus_tropicalis/Transcript/ProteinSummary?db=core;g=ENSXETG00000021646;r=scaffold_289:1213210-1217129;t=ENSXETT00000046776) | 1, 2, 3 |
| *Oryzias latipes* | Medaka TRYPSIN | **A4UWM7,** NP_001098370, **ENSORLP00000025702** | 1, 2, 3 |
| *Oryzias latipes* | Medaka CFD | [Q2Z1R4](http://www.uniprot.org/uniprot/Q2Z1R4), NP_001098200, [ENSORLP00000022520](http://www.ensembl.org/Oryzias_latipes/Transcript/ProteinSummary?db=core;g=ENSORLG00000017983;r=22:21883241-21980702;t=ENSORLT00000022521) | 1, 2, 3 |
| *Danio rerio* | Zebrafish TRYPSIN | [Q8AV83](http://www.uniprot.org/uniprot/Q8AV83), NP_571783, [ENSDARP00000063124](http://www.ensembl.org/Danio_rerio/Transcript/ProteinSummary?db=core;g=ENSDARG00000042993;r=16:23599477-23603992;t=ENSDART00000063125) | 1, 2, 3 |
| *Danio rerio* | Zebrafish CFD | [Q6DBS8](http://www.uniprot.org/uniprot/Q6DBS8), NP_001018368, [ENSDARP00000057824](http://www.ensembl.org/Danio_rerio/Transcript/ProteinSummary?db=core;g=ENSDARG00000039579;r=13:13735442-13738094;t=ENSDART00000057825) | 1, 2, 3 |
| *Danio rerio* | Zebrafish Chymotrypsin | Q7SX97, NP_997783, ENSDARP00000036127 | 1, 2, 3 |
| *Danio rerio* | Zebrafish PLG | Q6PBA6, NP_958880, ENSDARP00000045815 | 1, 2, 3 |
| *Tetraodon nigroviridis* | Pufferfish TRYPSIN | **Q4SH19,** CAG00063, [ENSTNIP00000012555](http://www.ensembl.org/Tetraodon_nigroviridis/Transcript/ProteinSummary?db=core;g=ENSTNIG00000009675;r=8:6007740-6009022;t=ENSTNIT00000012746) | 1, 2, 3 |
| *Tetraodon nigroviridis* | Pufferfish CFD | **Q4STJ9**, CAF96033, [ENSTNIP00000009528](http://www.ensembl.org/Tetraodon_nigroviridis/Transcript/ProteinSummary?db=core;g=ENSTNIG00000006744;r=10:12619215-12620588;t=ENSTNIT00000009703) | 1, 2, 3 |
|  |  |  |  |

*The URL for the databases are: ^1^UniProt: <http://www.uniprot.org/>;

^2^NCBI: <http://www.ncbi.nlm.nih.gov/>; ^3^ENSEMBL: <http://www.ensembl.org/>; release 55

^t^truncated
